# Lung endothelium exploits susceptible tumor cell states to instruct metastatic latency

**DOI:** 10.1038/s43018-023-00716-7

**Published:** 2024-02-02

**Authors:** Moritz Jakab, Ki Hong Lee, Alexey Uvarovskii, Svetlana Ovchinnikova, Shubhada R. Kulkarni, Sevinç Jakab, Till Rostalski, Carleen Spegg, Simon Anders, Hellmut G. Augustin

**Affiliations:** 1 European Center for Angioscience, Medical Faculty Mannheim, Heidelberg University, Heidelberg, Germany; 2 Division of Vascular Oncology and Metastasis, German Cancer Research Center Heidelberg (DKFZ–ZMBH Alliance), Heidelberg, Germany; 3 Faculty of Biosciences, Heidelberg University, Heidelberg, Germany; 4 Center for Molecular Biology, Heidelberg University, Heidelberg, Germany; 5 Evotec SE, Göttingen, Germany; 6 Bioquant Center, Heidelberg University, Heidelberg, Germany

**Keywords:** Cancer microenvironment, Metastasis, Metastasis, Cancer

## Abstract

In metastasis, cancer cells travel around the circulation to colonize distant sites. Due to the rarity of these events, the immediate fates of metastasizing tumor cells (mTCs) are poorly understood while the role of the endothelium as a dissemination interface remains elusive. Using a newly developed combinatorial mTC enrichment approach, we provide a transcriptional blueprint of the early colonization process. Following their arrest at the metastatic site, mTCs were found to either proliferate intravascularly or extravasate, thereby establishing metastatic latency. Endothelial-derived angiocrine Wnt factors drive this bifurcation, instructing mTCs to follow the extravasation–latency route. Surprisingly, mTC responsiveness towards niche-derived Wnt was established at the epigenetic level, which predetermined tumor cell behavior. Whereas hypomethylation enabled high Wnt activity leading to metastatic latency, methylated mTCs exhibited low activity and proliferated intravascularly. Collectively the data identify the predetermined methylation status of disseminated tumor cells as a key regulator of mTC behavior in the metastatic niche.

## Abstract (web summary)

Jakab et al. show that metastasizing tumor cells have a predetermined methylation status that allows them to respond differentially to endothelial cell niche-derived Wnt signals, resulting in either latency or intravascular proliferation.

## Main

Metastatic latency and tumor cell (TC) dormancy pose a major hurdle in the treatment of cancer^1–3^. During metastasis, latent TCs (LTCs) reside in close proximity to blood vessels and acquire a stem-like phenotype^4^. However, metastasizing tumor cells (mTCs) show remarkable heterogeneity in their genetic and molecular make-up, which can be attributed to certain cancer cells reaching a latent state whereas others outgrow immediately to form macrometastases^5–9^. This differential behavior is not solely driven by TC-intrinsic properties but is also influenced by this metastatic niche, because certain niches in principle favor TC proliferation whereas others are primarily tumor suppressive^10–12^. This argues for a scenario in which the induction of latency depends on cell-intrinsic properties and matching microenvironmental factors, which is also corroborated by the finding that disseminated TCs, once committed to a dormant fate, require dramatic events to be awakened^13–15^. We therefore hypothesized that TC behavior is primed during the initial arrival of mTCs at the metastatic niche and that the vascular endothelium, as the interface of dissemination, is a crucial fate instructor.

## Wnt and epithelial-to-mesenchyme transition pathways drive extravasation and latency

**Fig. 1:**
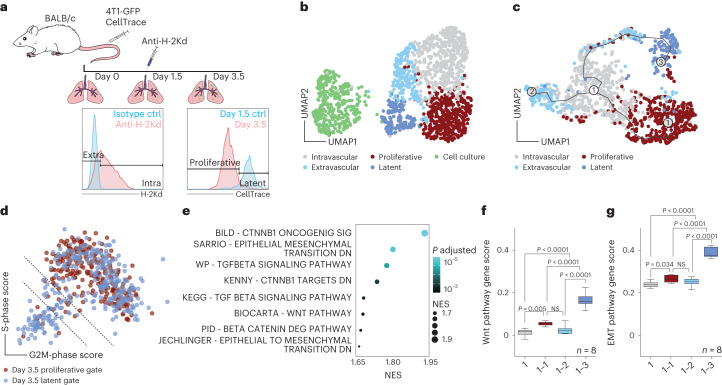
Extravasation and LTCs are defined by a Wnt and EMT signature. **a**, Schematic of the experimental design. Recipient BALB/c mice received two injections of 1 × 10^6^ 4T1-GFP cells stained with CellTrace dye via the tail vein. At day 1.5 postinjection, mice were injected intravenously with 5 µg of fluorescently labeled anti-H-2Kd antibody. TCs and ECs were sampled at day 0 (uninjected baseline), day 1.5 and day 3.5. TCs were discriminated based on both extravasation status on day 1.5 and proliferation status on day 3.5. **b**, UMAP of total TC dataset, with 1,556 cells passing quality control. SNN-based clustering resolved transcriptomes of TCs into five clusters; *n* = 4 mice per time point. **c**, Trajectory analysis of extracted TCs from combined day 1.5 and day 3.5 time points reveals three transition branches; the dataset contains 1,194 cells. **d**, Scatter plot of S- and G2M-phase gene expression scores for individual cells extracted on day 3.5 and colored by respective FACS gates. Dotted lines indicate thresholds of cells with score sums <−1 (lower line) and <0 (upper line). **e**, GSEA of genes upregulated in bona fide LTCs, ranked by fold change. Selected gene sets are shown (the full set is displayed in Supplementary Table 2). *P* values computed by permutation. **f**,**g**, Gene scores for Wnt pathway-associated genes (**f**, 146 genes) and for genes upregulated during EMT transition (**g**, 384 genes). Analysis of pseudobulks of manually selected TCs along the branches of the trajectory in **c** and grouped by biological replicate, with pseudobulks reflecting intravascular cells (1), cells on the intravascular–proliferative branch (1–1), cells on the intravascular–extravascular branch (1–2) and cells on the extravascular–latency branch (1–3). *P* values by one-way analysis of variance (ANOVA) with Tukey post test are shown. Box size represents interquartile range (IQR), with midline representing the median of the data, upper line the upper quartile and lower line the lower quartile. Whiskers represent 1.5 × IQR. Ctrl, control; NES, normalized enrichment score; NS, not significant. Source data

**Extended Data Fig. 1:**
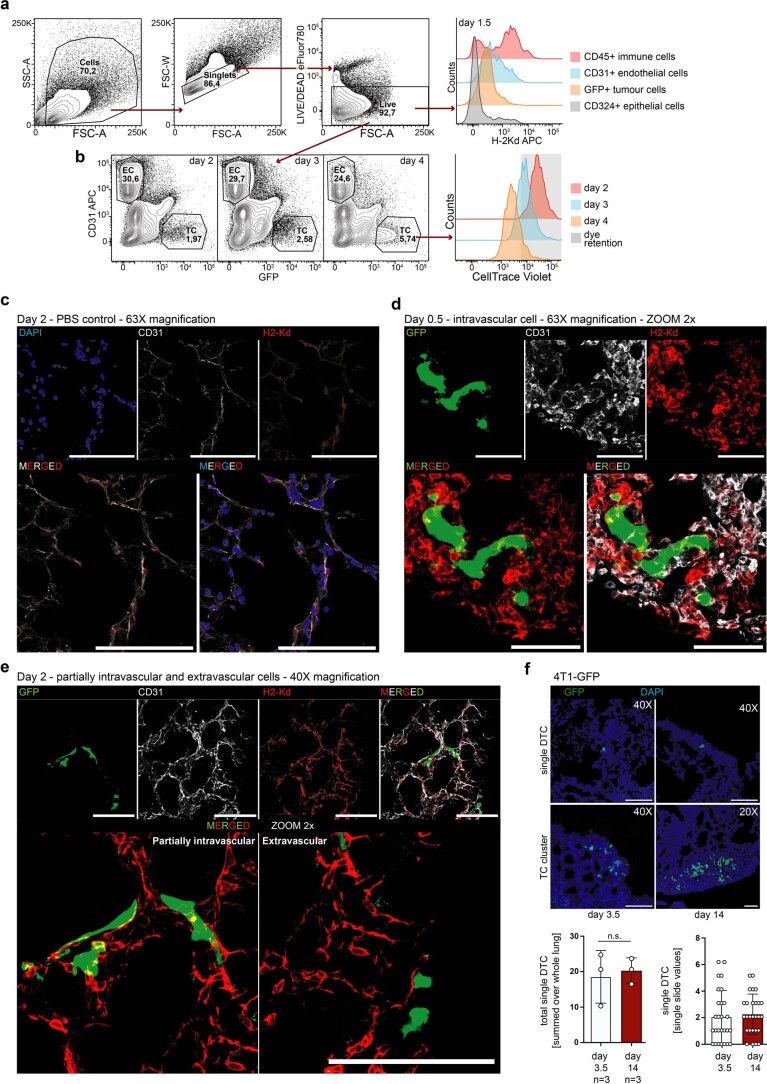
FACS-based gating strategy to enrich rare metastasising tumor cell sub-populations. **a**, Representative FACS-gates for purifying extravascular and intravascular cells using a self-validating system. Cells (immune and endothelial cells) that are anatomically positioned to be exposed to the circulation show staining, cells that are excluded from the circulation (epithelial cells, tissue resident immune cells) are not stained. TC show partial staining reflecting their extravasation status. **b**, Representative FACS plots of relative TC abundance in the lung two, three and four days postinjection (left panel) with corresponding temporal tracing of CellTrace dye dilution (right panel). Induction of TC proliferation occurs between day 3 and day 4 postinjection. TC with dye retention were considered latent. For day 3.5 and day 4 experiments, the day 1.5 timepoint was included to set the dye retention gate. **c**-**e**, Representative images for validating the intravascular staining using anti-H-2Kd antibody. For all experiments, mice were injected with 30µg anti-H-2Kd-APC antibody 2 min prior to euthanasia. Lungs were excised, fixed in PFA and cryo-embedded. For all images stacks of 20 µm were acquired using sequential scans and z-projections using maximum intensity are displayed. **c**, Lung from PBS-injected control animal. Upper left panel, DAPI staining is shown (cell nuclei, blue), upper mid panel displays CD31 staining (blood vessels, white) and upper right panel H-2Kd staining (red). Lower left panel shows overlay of CD31 and H-2Kd staining and lower right panel displays overlay of DAPI, CD31, and anti-H-2Kd staining. Scale bars = 100 µm. All images were acquired using 63X magnification objective. **d**, Lung from mouse 0.5 days postinjection of 4T1-GFP tumor cells (intravascular control). Upper left panel, GFP staining is shown (TC, green), upper mid panel displays CD31 staining (blood vessels, white) and upper right panel H-2Kd staining (red). Lower left panel shows overlay of GFP and H-2Kd staining and lower right panel displays overlay of GFP, CD31, and anti-H-2Kd staining. Yellow colour indicates overlap of GFP and H-2Kd signal. Scale bars = 50 µm. All images were acquired using 63X magnification objective, using zoom factor 2. **e**, Lung from mouse 2 days postinjection of 4T1-GFP tumor cells. Upper left panel, GFP staining is shown (TC, green), upper mid left panel displays CD31 staining (blood vessels, white), upper mid right panel H-2Kd staining (red), and upper right panel the overlay of GFP, CD31, and H-2Kd. Lower panel shows zoom in (factor 2) of GFP and H-2Kd overlay. Lower left panel displays partially extravasated tumor cells and lower right panel displays fully extravasated tumour cells. Yellow colour indicates overlap of GFP and H-2Kd signal. Scale bars = 100µm. All images were acquired using 40X magnification objective. **f**, Representative images and quantifications of single disseminated tumor cells (sDTC) and tumor cell clusters in mice 3.5 days or 14 days post 4T1-GFP injection. For all images stacks of 12 µm were acquired and z-projections using maximum intensity are displayed. Magnifications are indicated in the respective image. All images display overlays of DAPI (cell nuclei, blue) and GFP (TC, green). Upper panels show sDTC in lungs 3.5 days (left panel) and 14 days (right panel) postinjection, and lower panels show respective cell clusters. Left quantification shows total sDTC per lung and group, and right quantification shows sDTC number counted for each slide (sum of 2 sections). Data are presented as mean values +/− s.d., p value by two-tailed t-test is shown. n.s., not significant., n = 3 mice. Source data

**Extended Data Fig. 2:**
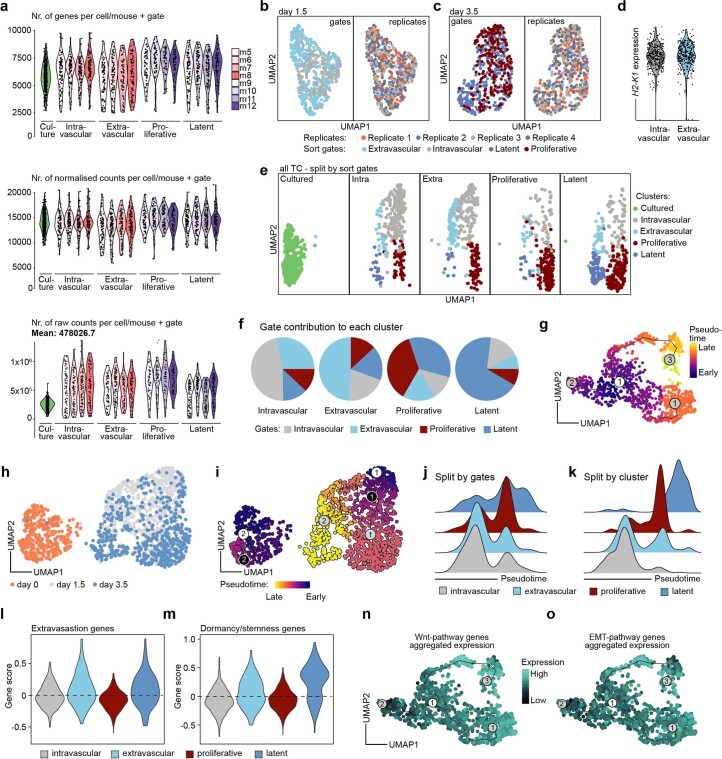
Characterisation of metastasising tumor cell sub-populations using scRNAseq. **a**, Number of detected genes (upper panel), normalized gene counts (mid panel) and total gene counts (lower panel) for each cell passing quality filtering split by FACS-gate and biological replicate. Only TC with detected genes >2500 were analysed. n = 4 mice. **b**, UMAP of TC extracted from lungs 1.5 days (632 cells) or **c**, 3.5 days postinjection (562 cells). Cells are coloured by FACS-gate (left panels) or by biological replicate (right panels). **d**, *H2-K1*-expression in individual TC isolated 1.5 days postinjection split by their respective FACS-gates. **e**, UMAP of all TC combined and split by the respective FACS-gates identifies cluster-enrichment for the sorted fractions. **f**, Pie charts displaying the contribution of the individual FACS gates to each cluster. Each pie chart represents one cluster, individual sections of the pie charts are coloured by FACS gates. **g**, Pseudo-temporal ordering of TC from the day 1.5 and day 3.5 timepoint combined. **h**, UMAP of all TC combined coloured by timepoint of sampling. **i**, Pseudo-temporal ordering of all TC combined. **j**, Ridge plots of the distribution of cells in pseudotime split by sort gates, and **k**, by cell clusters. **l**, Violin plots showing scored expression of genes associated with tumor cell extravasation, and **m**, of genes associated with tumor cell dormancy and cancer stemness in individual clusters of the TC scRNAseq dataset. **n**, Visualization of the aggregated expression of Wnt pathway-associated genes and **o**, genes upregulated during EMT on the trajectory. Source data

To test our hypothesis we developed an experimental model for temporal assessment of TC and endothelial cell (EC) interactions in the metastatic niche in vivo and at single-cell resolution. For this purpose, wild-type female BALB/c mice were intravenously injected with green fluorescent protein (GFP)-expressing 4T1 breast cancer cells (4T1-GFP). Lung-seeded TCs and corresponding total lung ECs were isolated at day 0 (baseline), day 1.5 (peak phase of TC extravasation) and day 3.5 (induction of TC proliferation) and enriched by fluorescence-activated cell sorting (FACS) (Fig. 1a and Extended Data Fig. 1a,b). Plate-based, single-cell RNA sequencing (scRNA-seq) was used to analyze the transcriptional signatures of TC–EC interactions during metastatic colonization. To discriminate extravascular from intravascular TCs, mice were intravenously injected at day 1.5 with fluorescently labeled anti-H-2Kd antibody. This antibody targets BALB/c-specific major histocompatibility complex I thereby labeling all body cells, including syngeneic 4T1-GFP cells that were exposed to the circulation, and hence creating a self-validating system. Cell types exposed to the circulation, such as circulating immune cells and ECs, showed positive staining in FACS whereas epithelial cells and tissue-resident immune cells remained unstained (Extended Data Fig. 1a). The approach was further validated using microscopy, which revealed that H-2Kd staining was confined to the vasculature and stained only TCs or parts thereof (for partially extravasated TCs) that were within the blood vessel (Extended Data Fig. 1c–e). Because TCs started to proliferate between days 3 and 4 postinjection (Extended Data Fig. 1b), they were further discriminated by their proliferation status at day 3.5 based on the dilution of CellTrace dye, independently of their extravasation status. TCs that retained CellTrace dye to the same extent as nonproliferative TCs from the day 1.5 time point were deemed latent whereas those with dye dilution, as evidenced by lower staining intensity in FACS, were considered proliferative (Fig. 1a and Extended Data Fig. 1b). Importantly, LTCs persisted in the lung for at least 2 weeks, indicative of a stable latent phenotype that resembled metastatic dormancy (Extended Data Fig. 1f). For each FACS-enriched TC subpopulation and matched ECs, equal cell numbers were sorted from at least three biological replicates. The cells in the dataset showed homogenous distribution for raw gene counts, normalized counts and detected genes (Extended Data Fig. 2a) and the replicates interlaced well in uniform manifold approximation and projection (UMAP) (Extended Data Fig. 2b,c), demonstrating the robustness of the experimental approach. Moreover, substructures in UMAP were found to be specifically enriched for cells from the respective TC FACS gates, suggesting that the gating strategy was suitable for enrichment of rare TC subpopulations even though it did not yield high purity (Extended Data Fig. 2b–f). Importantly, *H2-K1* expression did not differ between the intravascular and extravascular fractions, highlighting that differences in staining intensity reflect exposure to circulation rather than gene regulation (Extended Data Fig. 2d).

Clustering of the combined TC dataset identified a total of five clusters enriched for cells of the respective FACS gates (Fig. 1b and Extended Data Fig. 2e,f). Trajectory analysis to reconstruct the colonization process with TCs of the intravascular cluster set as starting point identified three main trajectories, transitioning (1) from intravascular to proliferative cells, (2) to a subset of extravascular cells and (3) through a subset of extravascular cells to LTCs (Fig. 1c). This also reflected the pseudotemporal ordering of events, with the establishment of latency occurring last, suggesting that proliferation had preceded extravasation and latency induction (Extended Data Fig. 2g). Importantly, pseudotime correlated with real time, indicating that the trajectory analysis had faithfully recapitulated the sequence of biological events (Extended Data Fig. 2h–k). Moreover, extravascular TCs showed enrichment of genes that were previously shown to be involved in the extravasation process^16^ while LTCs were enriched for genes associated with cancer stemness and dormancy (Extended Data Fig. 2l,m and Supplementary Table 1). Taken together, these findings indicated that TC extravasation is a prerequisite for metastatic latency but dispensable for TC proliferation, which molecularly defines earlier microscopy-based concepts^17–19^. Importantly these data do not rule out extravascular TC growth, albeit strongly suggesting that intravascular cells are the major contributor to metastatic growth in the lung.

We next compared proliferative versus latent cells and scored each TC from the day 3.5 dataset for the expression of G2M- and S-phase genes. To exclude cells that had proliferated but dropped out of cycle, only TCs that showed dye retention and were not in cycle at the time point of sampling were considered as proliferation naïve and labeled as bona fide latent (Fig. 1d). Following regression of cell cycle-associated genes, differential gene expression analysis (DGEA) between bona fide latent and proliferative TCs was performed with subsequent gene set enrichment analysis (GSEA) (Fig. 1e and Supplementary Table 2). In line with previous reports^20,21^, transforming growth factor beta-signaling and epithelial-to-mesenchyme transition (EMT) gene sets were enriched in latent mTCs, suggesting early priming for tumor dormancy. Surprisingly, β-catenin-mediated canonical Wnt signaling was identified as one of the most significantly enriched pathways (Fig. 1e). Wnt ligands have been extensively characterized as protumorigenic growth factors^22^, promoting proliferation in both primary tumors and metastases as well as circulating TC (CTC) survival^23–26^. Unexpectedly, the expression of Wnt pathway- and EMT-associated genes was enriched alongside the extravascular–latency trajectory (Fig. 1f,g, Extended Data Fig. 2n,o and Supplementary Table 1), supporting the hypothesis that niche-derived Wnt ligands may drive the metastatic latency of a subset of mTCs that is characterized by a mesenchymal-like phenotype. Overall these data provide a transcriptional blueprint of mTC fate decisions in the metastatic lung.

## Lung endothelium displays bimodal response following arrival of mTCs

**Extended Data Fig. 3:**
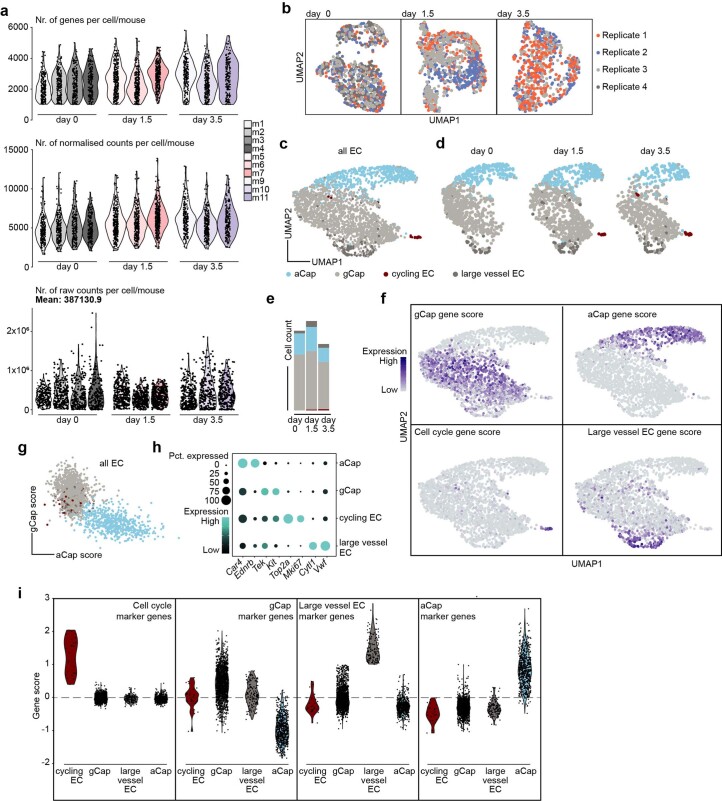
Classifying lung EC reveals emergence of cycling and activated gCap. **a**, Number of detected genes (upper panel), normalized gene counts (mid panel) or total gene counts (lower panel) for each cell passing quality filtering split by timepoint and biological replicate. Only EC with detected genes >1000 were analysed. n = 3-4 mice. **b**, UMAP of lung EC isolated at day 0 (left panel, 839 cells), day 1.5 (mid panel, 941 cells) and day 3.5 (right panel, 699 cells) coloured by biological replicate. n = 3-4 mice. **c**, UMAP of classified EC coloured by cell type (2,479 cells) and **d**, split by timepoint. **e**, EC cell type distribution split by timepoint. **f**, Visualization of marker gene scores used for classification of lung EC on the UMAP. **g**, Scatter plot of gCap vs. aCap gene scores in individual lung EC. Cells are coloured by classified cell type. **h**, Dot plot of selected EC subtype marker genes. Dot size reflects percentage of total EC that express the marker gene and color reflects expression level. **i**, Violin plots of marker gene scores used for cell classification split by classified EC subtype.

To assess the endothelium’s response towards arriving mTCs, ECs of the combined dataset were first classified into known lung-specific EC subtypes—that is, general capillary ECs (gCaps), aerocytes (aCaps), cycling ECs and large-vessel ECs—based on previously reported cell marker gene expression^27,28^ (Extended Data Fig. 3a–i and Supplementary Table 1). For this purpose, each EC was scored for the expression of the marker gene sets and thresholds were set for classification of EC type (Extended Data Fig. 3f–i).

**Fig. 2:**
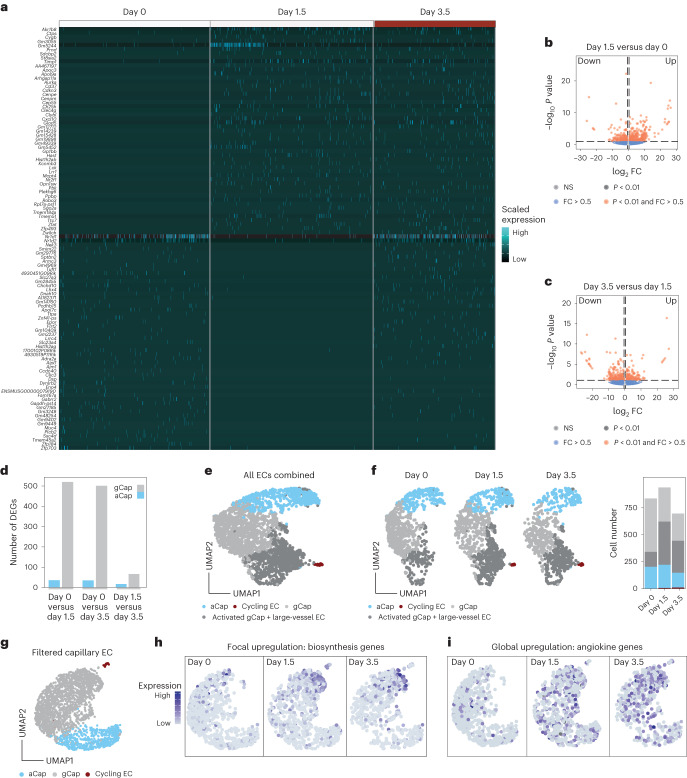
Lung endothelium exhibits an immediate bimodal response pattern. **a**–**c**, Differential gene expression analysis of lung EC pseudobulks comparing day 0 versus day 1.5 and day 1.5 versus day 3.5 using DESeq2. **a**, Heatmap of highly differentially expressed genes across the experimental timeline, with log_2_ fold change (FC) >3.5 and *P* <0.01, as computed in DESeq2 using the Wald test. Scaled expression is shown. **b**,**c**, Volcano plots of differentially expressed genes for comparison of day 1.5 versus day 0 (**b**; full set of DEGs shown in Supplementary Table 3) and for comparison of day 3.5 versus day 1.5 of total lung EC pseudobulks (**c**; full set of DEGs shown in Supplementary Table 4). FC and *P* values were computed in DESeq2 using the Wald test. Dotted lines represent thesholds of significance. Horizontal dotted lines indicate fold changes > |0.5|, vertical dotted lines indicate ***P*** values < 0.01. **d**, Differentially expressed genes were calculated using gCap and aCap pseudobulks comparing day 0 with days 1.5 and 3.5, as well as day 1.5 with day 3.5. Numbers of significant DEGs for each comparison and EC type are shown. DEGs were computed using the Wilcoxon rank-sum test. Genes were considered significant for log_2_ FC > 0.5 and *P* < 0.01. **e**, UMAP showing SNN-based clustering of total lung ECs resolved four cell clusters, which were annotated using the same marker genes for the classification shown in Extended Data Fig. 3. The activated gCap cluster was annotated based on upregulated genes compared with the gCap cluster (full set of DEGs shown in Supplementary Table 5). **f**, UMAP of total lung ECs split by time point and colored by cluster identity (left); barplot showing cell number per cluster and time point (right). The dataset contains 2,479 cells; *n* = 3–4 mice per time point. **g**, UMAP of filtered capillary lung ECs, colored by classified cell type. The dataset contains 2,293 cells. **h**,**i**, visualization of gene expression as gene scores in UMAP for the biosynthesis gene set (**h**) and angiokine gene set (**i**), split by time point.

Next, DGEA of EC pseudobulks comparing individual experimental time points was performed, which revealed an immediate response pattern in gCaps with genes being mostly regulated at day 1.5 whereas aCaps showed little transcriptional dynamics (Fig. 2a–f and Supplementary Tables 3 and 4). To investigate the molecular basis of the observed gCap response, clustering analysis was performed, which revealed the emergence of a gCap subpopulation that clustered together with large-vessel ECs (Fig. 2e,f). DGEA between the emerging gCap cluster and gCap revealed upregulation of mostly metabolic and ribosomal genes, indicating enhanced biosynthesis and general activation (Supplementary Table 5). Significantly regulated genes were further filtered for temporal enrichment (increased on days 1.5 and 3.5 compared with day 0), and the gene expression profile of the resulting gene panel (Supplementary Table 1) was investigated in filtered capillary ECs (Fig. 2g,h). Interestingly, capillary ECs showed focal expression and upregulation of the gene panel, suggesting a subset of metabolically active ECs that produce biomass following TC arrival.

It was previously established that the endothelium serves as a systemic amplifier of primary tumor-derived signals^29,30^. We therefore sought to determine whether a similar upregulation of immune-modulatory angiokines (that is, EC-derived cyto- or chemokines) could be observed in our dataset. For this purpose a gene panel of known angiokines was compiled (Supplementary Table 1) and their gene expression assessed, which revealed global and temporal upregulation of angiokines across all capillary ECs (Fig. 2i).

Collectively the data led us to conclude that the lung endothelium responds in a bimodal manner towards mTCs by focal production of biomass while exerting important immune-regulatory functions at the systems level.

## Angiocrine Wnt ligands instruct metastatic latency

**Fig. 3:**
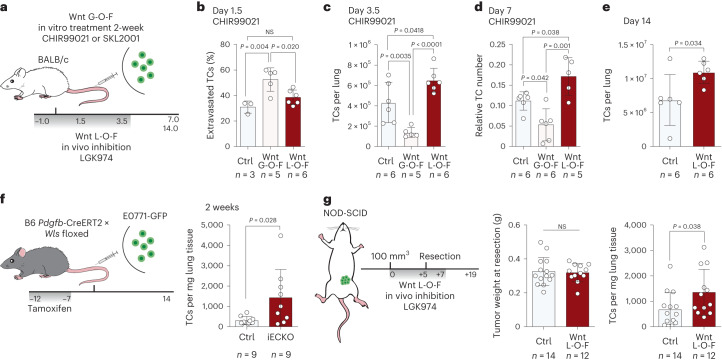
Lung endothelial cells are a major source of latency-inducing Wnt ligands. **a**, Schematic of the experimental G-O-F and L-O-F (loss-of-function) strategy. G-O-F was achieved by treatment with either CHIR99021, a selective GSK-3 inhibitor that leads to the accumulation of β-catenin via inhibition of its proteasomal degradation, or SKL2001, which directly interacts with β-catenin and thereby stabilizes the protein. L-O-F was achieved by treatment with LGK974, a selective inhibitor of Porcupine, an enzyme that is crucially involved in the secretion of Wnt ligands. Gray bar indicates time span (in days) of daily treatment with LGK974. **b**, Percentage of extravasated TCs for control, G-O-F and L-O-F 1.5 days postinjection. Data presented as mean ± s.d.; *P* values by one-way ANOVA with Tukey post test; *n* = 3–6 mice. **c**, Quantification of absolute TC number per lung 3.5 days postinjection for control, G-O-F and L-O-F. Data presented as mean ± s.d., *P* values by one-way ANOVA with Tukey post test; *n* = 5–6 mice. **d**, Quantification of relative TC number normalized to EC abundance in lungs 7 days postinjection for control, G-O-F and L-O-F. Data presented as mean ± s.d., *P* values by one-way ANOVA with Tukey post test; *n* = 5–6 mice. **e**, Quantification of absolute TC number per lung 14 days postinjection for control and L-O-F. Data presented as mean ± s.d., *P* value by two-tailed *t*-test; *n* = 6 mice. **f**, Schematic of experiment. Gene recombination was induced by tamoxifen administration: 2 × 10^5^ E0771-GFP cells were injected into the tail vein of EC-specific knockout (iECKO) and control animals. Gray bar indicates time span (in days) of daily tamoxifen treatment (left). Total number of TCs per mg lung tissue of control and iECKO mice 2 weeks postinjection of E0771-GFP (right). Data presented as mean ± s.d., *P* value by two-tailed *t*-test; *n* = 9 mice. **g**, Left, schematic of the experiment. 1 × 10^6^ 4T1-GFP cells were implanted into the mammary fat pad of NOD-SCID mice. Once tumors had reached a size of 100 mm³, mice were treated with LGK974 for 5 days until tumor resection. Following resection, mice were treated for an additional 2 days and left to develop metastases. Gray bar indicates time span (in days) of daily treatment with LGK974. Weights of resected primary tumors (middle) and total number of TCs per mg lung tissue of control and LGK974-treated mice 2 weeks postresection (right). Data presented as mean ± s.d., *P* value by two-tailed *t*-test; *n* = 12–14 mice. Source data

**Extended Data Fig. 4:**
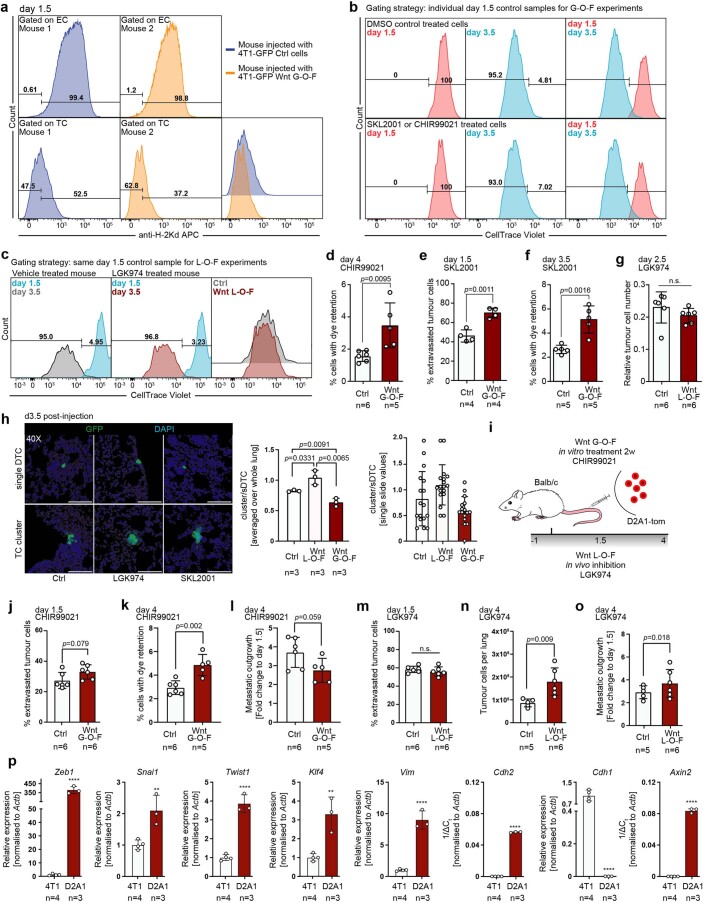
Niche-derived Wnt-ligands induce extravasation and latency in experimental metastasis models. **a-c**, Representative FACS gates for quantifications of extravasated and latent tumor cells. **a**, Gating strategy for discriminating extravascular from intravascular tumor cells are shown for a mouse injected with 4T1 control cells (mouse 1, dark blue line) and a mouse injected with 4T1 G-O-F cells (mouse 2, orange line). Gates were set for the control group so that all EC fall into the intravascular gate (upper left panel). The quality of the gating was assessed for each individual sample (upper right panel) and adjusted if grave differences were observed. Samples were excluded if cells were insufficiently stained. EC gates were pasted to tumor cells (lower panels) and the percentage of stained (intravascular) and unstained (extravascular) cells was measured. Lower panels show analysed control TC histograms (left panel), G-O-F TC histograms (mid panel), and the overlay (right panel). **b**, Gating strategy to quantify latent TC in G-O-F experiments. For each treatment group, one additional mouse was included in the experiment that was euthanized at day 1.5 and served as latent control. Latent gates were set in the respective day 1.5 control samples in a way that all TC fall into the latent gate. Gates were pasted to day 3.5 samples and percentage of latent TC was measured. Left panel shows histogram of day 1.5 TC (red line), mid panel show histogram of day 3.5 TC (blue line) and right panel shows overlay of day 1.5 and day 3.5 histograms for control treated (upper panels) and G-O-F tumor cells (lower panels). **c**, Representative gating for L-O-F experiment. The gating occurred in principle as outlined above, with the difference that only one day 1.5 control was included, as the same TC were injected for both groups. Left panel displays overlay of day 1.5 control histogram (blue line) and day 3.5 TC extracted from control treated mouse (grey line). Mid panel shows overlay of day 1.5 control histogram (blue line) and day 3.5 TC extracted from LGK974 treated mouse (red line). Right panel shows overlay of day 3.5 TC extracted from control (red line) and LGK974 treated mouse (grey line). **d**, Percentage of TC with dye retention 4 days postinjection of 4T1-GFP control and Wnt-agonist CHIR99021 long-term treated cells. n = 5-6 mice. **e**, Percentage of extravasated TC 1.5 days postinjection of 4T1-GFP control and Wnt-agonist SKL2001 long-term treated cells and **f**, Percentage of TC with dye retention at day 3.5. n = 4-5 mice. **g**, Relative tumor cell abundance in lungs of Ctrl treated and LGK974 treated mice 2.5 days postinjection of 4T1-GFP tumor cells. Relative tumor cell abundance was calculated as percentage of lung EC, which served as nonproliferative intra-sample control. n = 6 mice. **h**, Representative images and quantifications of single disseminated tumor cells (sDTC) and tumor cell clusters in mice 3.5 days postinjection for Ctrl (left panels), Wnt L-O-F (mid panels) and Wnt G-O-F (SKL2001) (right panels). For all images stacks of 12 µm were acquired and z-projections using maximum intensity are displayed. All images were acquired using 40X magnification. Panels display overlays of DAPI (cell nuclei, blue) and GFP (TC, green). Upper panels show sDTC and lower panels display cell cluster. Quantification shows cell cluster to sDTC ratios, which were calculated for each lung by summing cell clusters and sDTC across all analysed sections. Quantification to the right displays ratios for each analysed slide (sum of two sections) per group. n = 3 mice. **i**, Schematic of experiment. **j**, Percentage of extravasated TC 1.5 days postinjection of D2A1-tom control and Wnt-agonist CHIR99021 long-term treated cells. n = 6 mice. **k**, Percentage of TC with dye retention at day 3.5 and **l**, metastatic outgrowth calculated as fold change of tumor cell abundance compared to the mean tumor cell abundance for each treatment group at day 1.5. n = 5-6 mice. **m**, Percentage of extravasated TC 1.5 days postinjection of D2A1-tom in Ctrl and LGK974 treated mice. n = 6 mice. **n**, Tumor cell abundance in lungs of Ctrl treated and LGK974 treated mice 3.5 days postinjection of D2A1-tom cells, and **o**, metastatic outgrowth of D2A1-tom cells in Ctrl or LGK974 treated mice. n = 5-6 mice. **p**, Relative expression of key EMT-associated genes *Zeb1*, *Snai1*, *Twist1*, *Klf4*, *Vim*, *Cdh2*, *Cdh1* and canonical Wnt-downstream target *Axin2* in cultured 4T1 cells or D2A1 cells. Target gene expression was normalized to Actb and relative expression to 4T1 cells was calculated using the 2^-ΔΔCt^-method. For genes without detectable expression in 4T1 cells 1/ΔCt is displayed. **d-p** Data are presented as mean values +/− s.d., *p* value by two-tailed t-test are shown. n.s., not significant. * *p* value < 0.05, ** *p* value < 0.01, *** *p* value < 0.001, **** *p* value < 0.0001. n = 3-4 independent experiments. Source data

Next we analyzed the consequences of the Wnt signature in LTCs. For this purpose, 4T1-GFP cells were treated in vitro for 2 weeks with either the canonical Wnt pathway agonist CHIR99021 (GSK-3 inhibitor) or SKL2001 (β-catenin stabilizer) before injection in a gain-of-function (G-O-F) approach. Conversely, mice were treated with a Porcupine inhibitor (LGK974) to create a Wnt-deficient environment (Fig. 3a) and the metastatic behavior of TCs was monitored (Extended Data Fig. 4a–c). As expected, Wnt G-O-F programmed TCs to follow the extravasation–latency route, which resulted in enhanced extravasation at day 1.5 postinjection and higher incidence of LTCs, as evidenced by a higher percentage of cells in the respective flow cytometry enrichment gates, leading to an overall reduced short-term metastatic burden (Fig. 3b,c and Extended Data Fig. 4d–h). In contrast, Wnt depletion enhanced short-term metastatic outgrowth but did not affect extravasation (Fig. 3b,c). Intriguingly, the observed short-term phenotypes were found to be stable for up to 2 weeks, the longest period in which mice could be retained in the experiment as endpoint criteria were reached (Fig. 3d,e), even though treatment with Porcupine inhibitor was stopped at day 4 postinjection. Moreover, Wnt inhibition did not alter the initial survival of TCs at the metastatic site (Extended Data Fig. 4g). Collectively these data indicated an initial priming of mTCs by niche-derived Wnt factors that led to the acquisition of a stable, latent phenotype.

Importantly, these observations were not cell type specific because repeating the experiments using dtTomato^+^ D2A1 (D2A1-tom) cells showed enhanced, yet not significant, extravasation following 2 weeks of in vitro treatment with CHIR99021, which consequently resulted in a higher fraction of LTCs and a reduced short-term metastatic burden (Extended Data Fig. 4i–l). Similarly, depletion of Wnt in mice did not affect the extravasation of D2A1-tom cells but enhanced short-term metastatic burden and outgrowth (Extended Data Fig. 4m–o). Curiously, while the effect of Wnt depletion was similar between D2A1 and 4T1 cells, the phenotypes of the G-O-F experiment were considerably weaker. To investigate this divergence, the gene expression profile of well-established EMT and canonical Wnt pathway target genes was assessed in D2A1 and 4T1 cell lines. To our surprise, D2A1 cells were of mesenchymal character and showed baseline canonical Wnt-signaling activity (Extended Data Fig. 4p), which may explain the mitigated effect observed for the Wnt G-O-F experiment.

**Extended Data Fig. 5:**
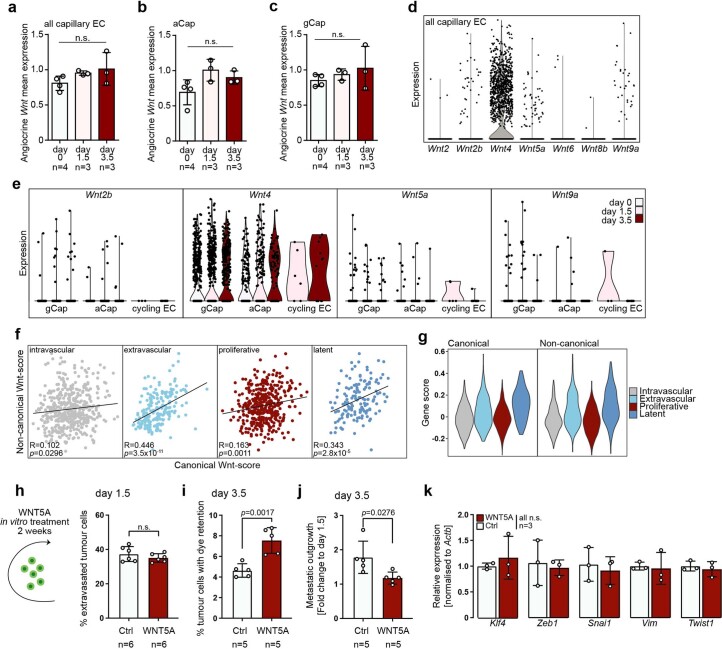
Lung endothelial cells express canonical and noncanonical Wnt ligands. **a**, Mean summed expression of Wnt-ligands in all capillary EC, **b**, in filtered aCap or **c**, in filtered gCap pseudo-bulks of biological replicates. Data are presented as mean values +/− s.d., *p* values were calculated by one-way ANOVA with Tukey post-test. n.s., not significant. n = 3-4 mice. **d**, Expression profile for detected Wnt-ligands in individual lung EC of the combined scRNAseq dataset. **e**, Expression profile of the 4 top expressed Wnt ligands split by timepoint and by EC subtype. **f**, Correlation of noncanonical Wnt gene scores and canonical Wnt gene scores in cells of the individual tumor cell clusters are shown. *P* value and *r* value by Pearson correlation are shown. **g**, Violin plots of scored gene expression of genes associated with canonical Wnt-signalling (left panel), and noncanonical Wnt-signalling (right panel) in individual TC clusters. **h**, Schematic of experiment. TC were treated 2 weeks *in vitro* with WNT5A or PBS control. Quantification shows percentage of extravasated tumor cells 1.5 days postinjection of control or WNT5A treated TC. n = 6 mice. **i**, Percentage of latent tumor cells 3.5 days postinjection of control or WNT5A treated TC, and **j**, metastatic outgrowth, calculated as fold change in TC abundance at day 3.5 compared to the mean of the respective day 1.5 timepoint. n = 5 mice. **k**, Relative expression of key EMT-associated genes *Klf4*, *Zeb1*, *Snai1*, *Vim*, and *Twist1* in cultured 4T1 control or WNT5A treated cells. Target gene expression was normalized to *Actb* and relative expression to 4T1 cells was calculated using the 2^-ΔΔCt^-method. n = 3 independent experiments. **h-k**, Data are presented as mean values +/− s.d., p value by two-tailed t-test are shown. n.s., not significant. Source data

Because modulation of Wnt signaling was sufficient to alter TC behavior in vivo, we probed for sources of Wnt ligands in the metastatic niche. The endothelium was found to robustly express several Wnt ligands across the experimental timeline. Given the promiscuous nature of the Wnt-signaling pathway, the combined expression of Wnt ligands in lung capillary ECs and specific vascular beds (aCap and gCap) was assessed. Surprisingly, Wnt was stably expressed throughout the experimental timeline (Extended Data Fig. 5a–c). Moreover, the expression of individual, detectable Wnt ligands was also not changed in either a time-dependent or EC subtype-specific manner (Extended Data Fig. 5d,e). Given that lung ECs expressed both canonical and noncanonical Wnt ligands (Extended Data Fig. 5d), the contribution of the respective pathways to the enriched Wnt gene signature in extravascular–latent TCs was examined. Extravascular and LTCs were enriched for noncanonical and canonical Wnt pathway-specific genes (Supplementary Table 1), indicating synergistic signaling (Extended Data Fig. 5f,g). We therefore tested whether noncanonical Wnt pathway activation would have an effect similar to that seen for canonical pathway activation. For this, 4T1-GFP cells were treated for 2 weeks in vitro with recombinant WNT5A and metastatic behavior was assessed in vivo. WNT5A treatment did not affect TC extravasation but limited metastatic outgrowth through induction of latency (Extended Data Fig. 5h–j). Moreover, WNT5A treatment did not induce EMT in vitro (Extended Data Fig. 5k), suggesting that canonical Wnt signaling may drive TC extravasation whereas latency is induced by both pathways.

**Extended Data Fig. 6:**
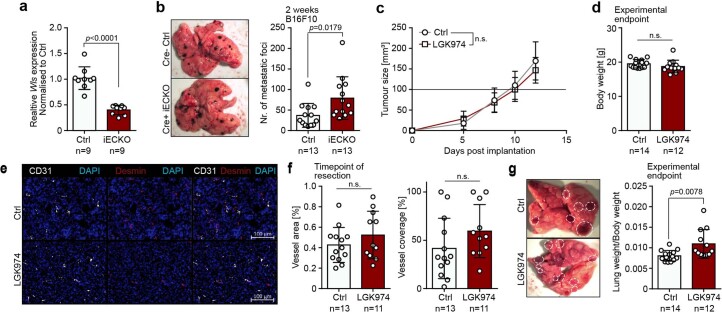
Angiocrine Wnt is a crucial instructor of metastatic latency. **a**, Recombination efficiency of Wls-iECKO mice. Expression of *Wls* was normalized to *Actb* and fold changes were calculated using the 2^-ΔΔCt^-method. Data are presented as mean values +/− s.d., *p* value by two-tailed t-test is shown. n = 9 mice. **b**, Representative images of metastatic lungs from Cre- control (upper image) and Cre+ iECKO mice (lower image) two weeks postinjection of 2 × 10^5^ B16F10 melanoma cells (left panel) and corresponding quantification of visible metastatic foci per lung (right panel). Data are presented as mean values +/− s.d., *p* value by two-tailed t-test is shown. n = 13 mice. **c**, Primary tumor growth curves for vehicle control and LGK974 treated mice. n = 14 for control group, n = 12 for LGK974 treatment group. Data are presented as mean values +/− s.d., *p* values for each measurement timepoint were calculated by two-tailed t-test. n.s., not significant. **d**, Body weights of mice at the experimental endpoint. Data are presented as mean values +/− s.d., *p* value was calculated by two-tailed t-test. n.s., not significant. n = 12–14 mice. **e**, Representative images of primary tumor vasculature for vehicle control (upper panels) and LGK974 treated mice (lower panels). **f**, Corresponding quantifications of total vessel area per region of interest (left panel) and desmin coverage of CD31+ vessels (right panel). Data are presented as mean values +/− s.d., *p* values were calculated by two-tailed t-test. n.s., not significant. n = 11–13 mice. **g**, Representative images (left panel) of metastatic lungs from vehicle control (upper image) and LGK974 treated mice (lower image). Dotted white circles indicate metastatic nodules. Quantification of lung weight to body weight ratio for vehicle control and LGK974 treated mice at the experimental endpoint. Data are presented as mean values +/− s.d., *p* value by two-tailed t-test is shown. n = 12–14 mice. Source data

Given that endothelial Wnt expression was not found to be altered across the experimental timeline (Extended Data Fig. 5a–e), the contribution of angiocrine Wnt in the establishment of latency was investigated. Depletion of Wnt ligands specifically from the vascular niche by EC-specific knockout (KO) of the Wnt cargo receptor *Wntless* (*Wls*) (Fig. 3f and Extended Data Fig. 6a) led to a significantly increased metastatic burden in experimental metastasis models, thereby phenocopying systemic pharmacological inhibition. This was observed for E0771-GFP breast cancer cells, which were used to avoid rejection of BALB/c-derived 4T1 cells in *Wls*-KO mice, but also for B16F10 melanoma cells (Fig. 3f and Extended Data Fig. 6b), indicating a more general mechanism and highlighting the endothelium as a major source of latency-inducing Wnt ligands. The importance of Wnt in priming of metastatic TCs for latency was also observed in a clinically relevant, spontaneous metastasis model involving surgical removal of the primary tumor. Specifically, 4T1-GFP cells were orthotopically implanted into the mammary fat pad of NOD-SCID mice to avoid GFP immunogenicity. Once tumors were established, mice were treated with Porcupine inhibitor until tumor resection. To ensure that all CTCs had reached a Wnt-deficient lung niche, mice were treated for two additional days postresection and left to develop overt metastases (Fig. 3g). Importantly, systemic treatment with LGK974 did not affect primary tumor growth and, hence, neither primary tumor size at the time point of resection, mouse body weight nor tumor vasculature (Fig. 3g and Extended Data Fig. 6c–f). However, Wnt depletion resulted in a significantly increased metastatic burden similar to that observed for the experimental metastasis models (Fig. 3g and Extended Data Fig. 6g), reinforcing the hypothesis of early Wnt-dependent priming of mTCs. Collectively, these data establish that homeostatic angiocrine Wnt ligands are crucial instructors of extravasated TC latency by priming of mTCs during their initial arrival at the metastatic lung niche.

## mTC behavior is predominantly driven by intrinsic features

**Fig. 4:**
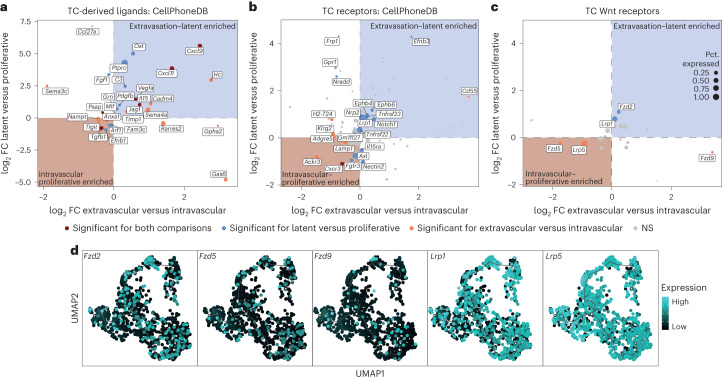
Resolving the temporal Wnt interactome of endothelial and TCs. **a**–**c**, DGEA on TC pseudobulks reflecting the sort gate and biological replicate. Extravascular cells were compared with intravascular cells and LTCs with proliferative cells (full set of DEGs is shown in Supplementary Table 6). DEGs were mapped against CellPhoneDB and filtered for genes that had annotated interaction partners expressed in the lung EC scRNA-seq dataset. *Y* axis shows log_2_ FC of DEGs for the latent versus proliferative comparison and *x* axis shows the log_2_ FC of the extravascular versus intravascular comparison. Blue and red backgrounds indicate enrichment of the gene for the extravasation–latent and intravascular–proliferative trajectory, respectively. Dot color represents significance: red indicates genes significantly differentially expressed for both latent–proliferative and intravascular–extravascular comparison; blue indicates genes significantly differentially expressed for latent–proliferative but not intravascular–extravascular comparison; and orange indicates genes that are significantly differentially expressed only for intravascular–extravascular comparison. Dot size indicates percentage of TCs with detectable gene expression in the scRNA-seq dataset (Pct. expressed). **a**, Annotated differentially expressed TC-derived ligands. **b**, Annotated differentially expressed TC receptors. **c**, Supervised analysis of Wnt receptors expressed in TCs. log_2_ FC and *P* values were computed in DESeq2 using the Wald test. Genes with adjusted *P* < 0.05 were considered significant. **d**, Expression pattern of differentially expressed Wnt receptors on the trajectory graph.

Because the expression of angiocrine Wnt ligands was not changed (Extended Data Fig. 5a–e), we reasoned that differences in Wnt signaling activity may have resulted from distinct TC receptor repertoires between latent and proliferative TCs. For this purpose, EC–TC interactions were predicted using CellPhoneDB^31^. To enhance the statistical robustness of the approach, TC pseudobulks were formed, reflecting the TC phenotype and the respective biological replicates. DGEA was performed comparing intravascular versus extravascular TCs and proliferative versus latent TCs to identify trajectory-defining interaction partners (Supplementary Table 6), which would be enriched in both comparisons (for example, upregulated in extravasated and LTCs). The resulting differentially expressed genes (DEGs) were mapped to CellPhoneDB^31^ and filtered for expression of the interaction partner in the EC dataset. Surprisingly, while this approach yielded several trajectory-defining TC-derived ligands and receptors, none of them belonged to the Wnt pathway (Fig. 4a,b). Repeating the analysis in a supervised manner by specific selection of TC-expressed Wnt receptors led to the identification of five (co)receptors that were differentially expressed for either the intravascular versus extravascular or latent versus proliferative comparison, but not for both (Fig. 4c). Moreover, receptor expression was enriched for neither the intravascular–proliferative nor extravasation–latency branch of the trajectory (Fig. 4d), suggesting that the observed differences in Wnt signaling activity were not established at the receptor ligand level.

**Fig. 5:**
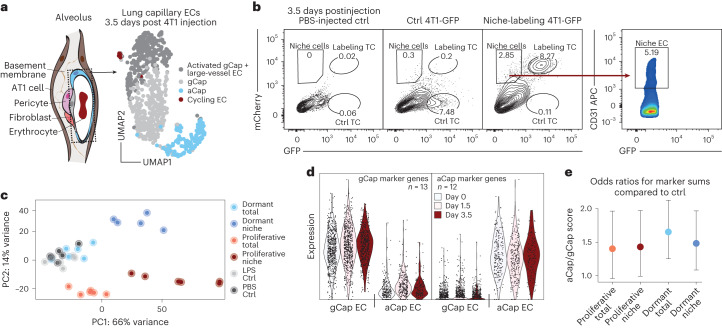
LTCs do not occupy distinct vascular niches in the lung. **a**, Left, schematic of lung alveolus; dotted box highlights ECs. Right, UMAP of EC transcriptomes reflecting the composition of bulk EC samples 3.5 days postinjection of 4T1-GFP cells. **b**, Representative FACS gates for purification of labeled niche ECs 3.5 days postinjection of niche-labeling 4T1-GFP cells. APC, allophycocyanin. **c**, Principal component (PC) analysis of samples included in the experiment. Total samples refer to unlabeled CD31^+^ ECs, niche samples refer to labeled CD31^+^ ECs, dormant samples refer to injections of niche-labeling D2.0R-GFP, proliferative samples refer to injections of niche-labeling 4T1-GFP. LPS control mice were injected intraperitoneally with LPS 24 h before euthanasia; PBS control mice were injected intravenously with PBS 3.5 days before euthanasia (full set of DEGs is shown in Supplementary Table 7); *n* = 5–6 replicates per condition. **d**, Summed expression of gCap and aCap marker genes in individual ECs split by both time point and EC identity. **e**, log_2_ FC of aCap/gCap marker genes odds ratios normalized to PBS-injected control samples. Dots represent mean of *n* = 5–6 replicates. Error bars indicate 95% confidence interval.

Because the lung endothelium harbors two distinct vascular beds that are defined by less penetrable gCaps, which are covered by pericytes and contain thick basement membranes and more traversable aCaps^27,28^ that lack pericyte coverage and have thin membranes (Fig. 5a), we tested whether distinct vascular niche occupancy could drive the observed differential TC behavior. Employing an in vivo niche-labeling system that enables the labeling of tissue cells that are in immediate physical proximity to a labeling donor cell through the secretion of a lipid-soluble mCherry variant^32^, we specifically enriched for TC-interacting lung ECs. Given that 4T1-GFP cells are mostly proliferative (>95% of cells with dye dilution at day 3.5), the dormant D2.0R-GFP breast cancer cell line was used as a proxy for LTCs. Labeled TC-interacting ECs, as well as matched, unlabeled total ECs, were FACS purified and subjected to bulk RNA-seq (Fig. 5b,c). To test whether dormant D2.0R cells in comparison with proliferative 4T1 cells preferably occupy the aerocyte niche, which could facilitate extravasation and induction of dormancy, the cellular composition of the bulk samples was deconvoluted. For this, aCap- and gCap-specific gene panels were assembled that showed robust and stable expression across the experimental timeline (Fig. 5d and Supplementary Table 1). The gene panels were then used to calculate an aCap- to gCap-specific transcript ratio for each sample, reflecting the relative abundance of aCap compared with gCap in the bulk sample. While a general bias towards the aCap signature could be observed for all tumor-bearing samples compared with PBS-injected control samples, no differences were detected between dormant and proliferative niche samples or their unlabeled counterparts (Fig. 5e), indicating that dormant TCs and proliferative TCs grossly occupy the same vascular niches in the lung.

**Extended Data Fig. 7:**
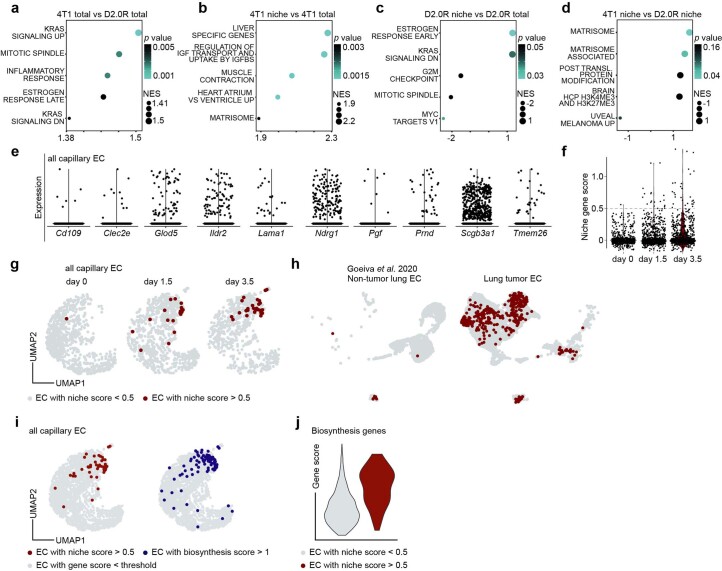
Proliferative niche endothelial cells promote matrix remodelling of the micro-niche. **a-d**, Gene set enrichment analyses of ranked upregulated genes. NES, normalized enrichment score, *p* values were computed by permutation. n = 6 mice per group. **a**, Gene sets enriched in unlabelled total EC from 4T1-injected lungs compared to unlabelled total EC from D2.0R-injected lungs. **b**, Gene sets enriched in labelled EC from 4T1-injected lungs compared to matched unlabelled total EC. **c**, Gene sets enriched in labelled EC from D2.0R-injected lungs compared to matched unlabelled total EC. **d**, Gene sets enriched in labelled EC from 4T1-injected lungs compared to labelled EC from D2.0R-injected lungs. **e**, Expression of proliferative niche EC markers genes in individual capillary EC from the combined scRNAseq dataset (full set of filtered marker genes for all conditions are displayed in Supplementary Table 8). **f**, Gene scores for marker genes in **e** for individual EC from the combined scRNAseq dataset split by experimental timepoint. Dotted line indicates threshold of EC with niche gene score > 0.5. **g**, Localisation of predicted tumor cell-interacting EC in the capillary EC dataset split by timepoint. **h**, Presence of predicted tumor cell-interacting EC in control non-tumorous and primary lung tumor EC in a publicly available dataset (13,854 cells). **i**, Localisation of predicted tumor cell-interacting EC and biosynthetic EC identified in Extended Data Fig. 4 in the combined capillary EC dataset. **j**, Violin plot of biosynthesis gene scores in endothelial cells identified as proliferative niche EC (niche score > 0.5, red colour) and non-niche EC (niche score < 0.5, grey colour).

Differential gene expression analysis of bulk lung EC samples (Supplementary Table 7) revealed that, in particular, proliferative TCs induced the production of extracellular matrix, an immune response program and proliferation in lung ECs. While matrix-remodeling processes were specifically enriched in the metastatic microniche, proliferative and proinflammatory programs were part of a systemic response towards the tumor challenge (Extended Data Fig. 7a–d). Matrix remodeling occurred predominantly in the proliferative niche (Extended Data Fig. 7d), indicating the notion that TCs, once committed to proliferation, actively change their microenvironment as part of a self-feeding forward loop. Because ECs in the proliferative tumor niche showed transcriptomic distinction, we sought to deduce a marker gene set allowing us to probe for proliferative TC-interacting ECs in our single-cell dataset. By testing all conditions against each other, including lipopolysaccharide (LPS)-injected control animals to avoid picking up general immune response genes, a gene panel specific for ECs extracted from the 4T1 niche was defined (Extended Data Fig. 7e and Supplementary Table 8). The resulting gene panel was used to predict tumor-interacting ECs in the scRNA-seq data by scoring the expression of the gene panel for each capillary EC. As expected, proliferative TC-interacting ECs emerged specifically at the day 1.5 and 3.5 time points and showed a marked increase for day 3.5 coinciding with the induction of TC proliferation (Extended Data Fig. 7f,g). To test the generality of the gene panel, a publicly available scRNA-seq dataset^33^ was utilized and similar enrichment was found specifically for primary lung tumor ECs compared with nontumorous matched samples (Extended Data Fig. 7h). Intriguingly, the predicted tumor-interacting ECs colocalized with previously identified biosynthetic ECs in the UMAP and were enriched for the expression of biosynthesis genes (Extended Data Fig. 7i,j), confirming our previous finding of a bimodal endothelial response towards arriving 4T1 TCs that includes systemic immunomodulatory function and biomass production in the physical microniche.

## Heterogenous methylation states predetermine mTC behavior

**Fig. 6:**
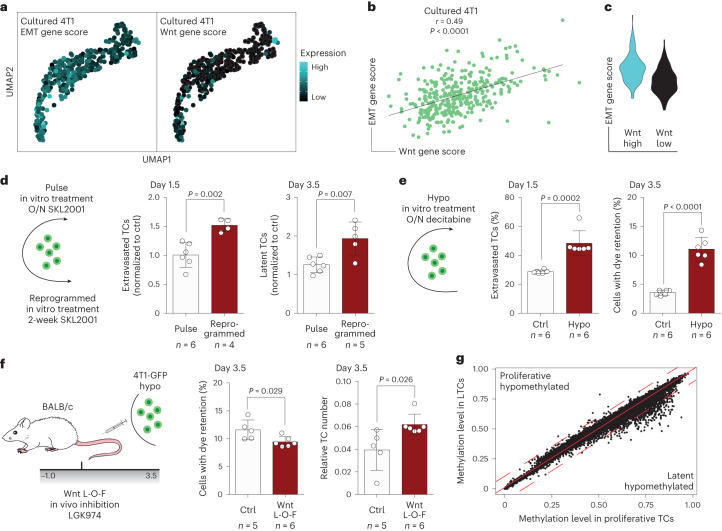
TC behavior in the metastatic niche is predetermined by methylation state. **a**, Gene scores of EMT (left)- and Wnt pathway-associated genes (right) in 362 cultured TCs visualized in UMAP. **b**, Correlation of gene scores. *P* and *r* values by Pearson correlation. **c**, Violin plot of EMT gene scores in cultured TCs with Wnt gene score >0 (Wnt high, cyan) and Wnt gene score <0 (Wnt low, black). **d**, Left, schematic of experiment. 4T1-GFP cells were either treated overnight (O/N) with Wnt agonist (pulse) or for 2 weeks (reprogrammed) before injection. Relative fraction of extravasated TCs normalized to the respective control 1.5 days postinjection (middle) and relative fraction of LTCs normalized to the respective control 3.5 days postinjection (right). Data presented as mean ± s.d., *P* values by two-way ANOVA with Sidak post test; *n* = 4–6 mice. **e**, Left, schematic of experiment. 4T1-GFP cells were treated overnight with the demethylating agent decitabine. Percentage of extravasated TCs for control and hypomethylation (hypo) treatment 1.5 days postinjection (middle) and percentage of latent TCs 3.5 days postinjection (right). Data presented as mean ± s.d., *P* values by two-tailed *t*-test; *n* = 6 mice. **f**, Left, schematic of experimental L-O-F approach. 4T1-GFP cells were treated overnight with decitabine. Gray bar indicates time span (in days) of daily treatment with LGK974. Percentage of latent TCs 3.5 days postinjection for control and LGK974-treated animals (middle) and relative TC number normalized to EC abundance in lungs (right). Data presented as mean ± s.d., *P* values by two-tailed *t*-test; *n* = 5–6 mice. **g**, Scatter plot of methylation level (fraction of methylated CpG islands) for gene bodies in latent and proliferative TCs. Solid red line indicates no differences in methylation, dotted red lines indicate thresholds for >10% differences in methylation. Source data

**Extended Data Fig. 8:**
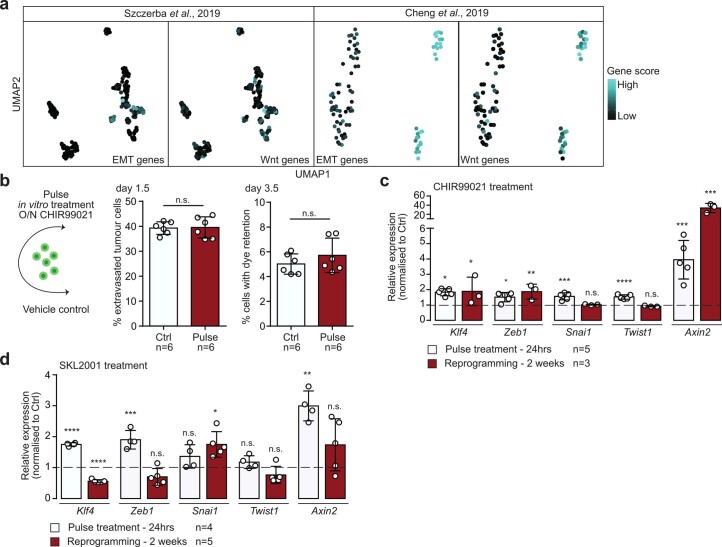
Wnt-agonistic treatment in vitro does not change cellular identity. **a**, EMT- and Wnt-gene scores in CTC isolated from breast cancer patients in publicly available datasets. Left panels display 252 cells, right panels display 89 cells. **b**, Schematic of experiment. 4T1-GFP cells were pulse treated with CHIR99021 or vehicle control overnight prior to injection (left panel). Percentage of extravasated TC 1.5 days (mid panel) and percentage of TC with dye retention 3.5 days postinjection (right panel). Data are presented as mean values +/− s.d., *p* values were calculated by two-tailed t-test. n.s., not significant. **c**, Relative expression of key EMT-inducing transcription factors *Klf4*, *Zeb1*, *Snai1* and *Twist1* and canonical Wnt-downstream target *Axin2* in 4T1 cells pulse-treated (over-night) or reprogrammed (two-week treatment) with Wnt-agonist CHIR99021 or **d**, SKL2001. Target gene expression was normalized to *Actb* and relative expression to respective controls was calculated using the 2^-ΔΔCt^-method. Data are presented as mean values +/− s.d., *p* value by two-tailed t-test are shown. n.s., not significant. * *p* value < 0.05, ** *p* value < 0.01, *** *p* value < 0.001, **** *p* value < 0.0001. Source data

**Extended Data Fig. 9:**
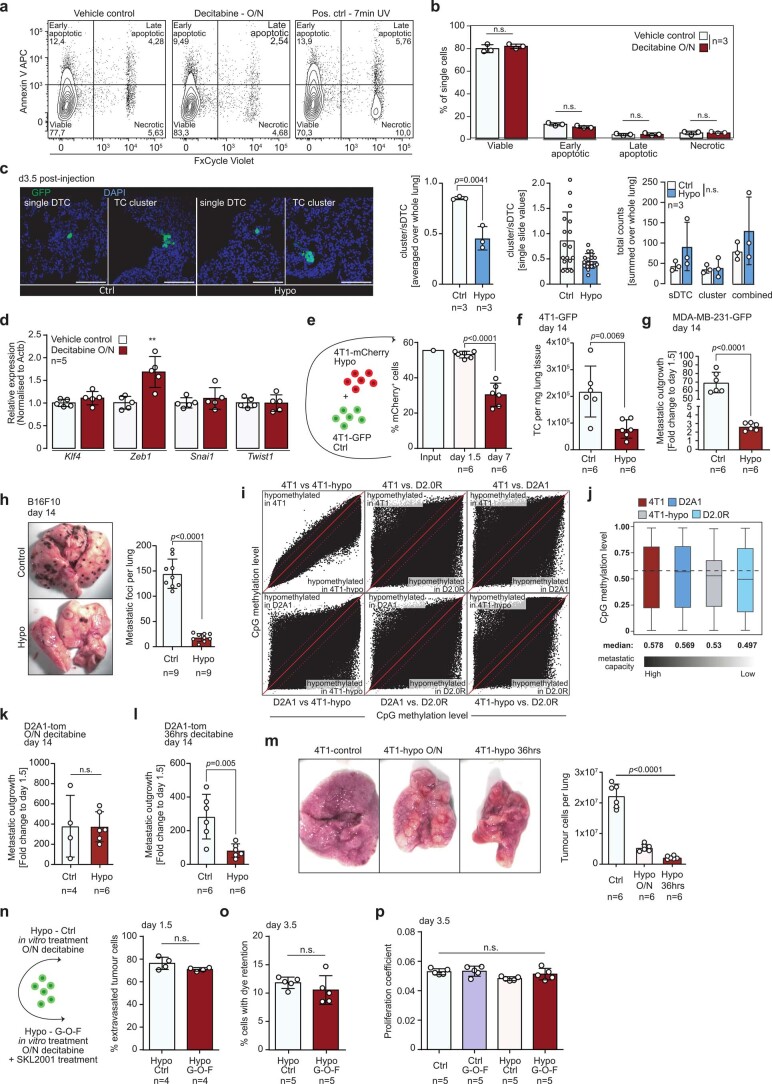
Hypomethylation does not affect cellular fitness or homing capacity but limits metastatic outgrowth. **a**, Representative flow cytometry gates for assessing cell viability of vehicle control treated (left panel), hypomethylated (mid panel) and UV irradiated positive control cells (right panel). **b**, Corresponding quantification of viable, early apoptotic, late apoptotic and necrotic vehicle control or overnight decitabine treated 4T1 cells Data are presented as mean values +/− s.d., *p* values were calculated by two-tailed t-test shown. n.s., not significant. n = 3 independent experiments. **c**, Representative images and quantifications of sDTC (left panels) and tumor cell clusters (right panels) in mice 3.5 days postinjection for Ctrl (two left images), and hypomethylated TC (two right images). For all images stacks of 12 µm were acquired and z-projections using maximum intensity are displayed. All images were acquired using 40X magnification. Panels display overlays of DAPI (cell nuclei, blue) and GFP (TC, green). Quantification shows cell cluster to sDTC ratios (left panel), which were calculated for each lung by summing cell clusters and sDTC across all analysed sections. Quantification in the middle displays ratios for each analysed slide (sum of two sections). Quantification on the right displays absolute counts per biological replicate for sDTC, tumor cell clusters and the combination of both. In order to adhere to the 3R principles, this experiment used the same controls and the same representative control images as the experiment presented in Extended Data Fig. 5h (see methods for specifications). n = 3 mice. **d**, Relative expression of key EMT-inducing transcription factors *Klf4*, *Zeb1*, *Snai1* and *Twist1* in 4T1 cells treated over-night with decitabine or vehicle control. Target gene expression was normalized to *Actb* and relative expression to respective controls was calculated using the 2^-ΔΔCt^-method. Data are presented as mean values +/− s.d., *p* value by two-tailed t-test is shown. ** *p* value < 0.01. n = 5 independent experiments. **e**, Schematic of experiment. 4T1-GFP control cells were mixed with overnight decitabine treated 4T1-mCherry cells in 1:1 ratio and injected into the tail vein of mice (left panel). Quantification of mCherry+ TC within the total TC pool at the timepoint of injection, 1.5 days and 7 days postinjection (right panel). Data are presented as mean values +/− s.d., *p* value by two-tailed t-test is shown. n = 6 mice. **f-h**, Cells were treated over-night with decitabine and metastatic burden was assessed 14 days postinjection for, **f**, 4T1-GFP cells (Balb/c mice) measuring tumor cell abundance per mg lung tissue, **g**, human MDA-MB-231-GFP breast cancer cells (NSG mice) measuring metastatic outgrowth calculated as fold change to day 1.5 injected control mice, and, **h**, B16F10 melanoma cells (in B6 mice), counting metastatic foci per lung. Representative images display whole metastatic lungs of mice injected with B16F10 control cells (upper image) or B16F10-hypo cells (lower image). Data are presented as mean values +/− s.d., *p* values by two-tailed t-test are shown. To adhere to the 3R principles, the experiment in panel **f** used the same control as the experiment presented in Fig. 2e (see methods for specifications). n = 6–9 mice. **i**, Box plots showing methylation level of CpGs included in the methyl array in 4T1, D2A1, 4T1-hypo and D2.0R cells. Black gradient below indicates metastatic capacity of the individual cell line, with 4T1 cells being the most aggressive and D2.0R cells being the least aggressive cell line. Size of box represents interquartile range (IQR), with midline representing the median of the data, upper line the upper quartile and lower line the lower quartile. Whiskers represent 1.5 times IQR. **j**, Scatter plot of methylation level for individual CpGs included in the methyl array comparing each cell line with each other. Red line indicates no differences in methylation, dotted red lines indicate thresholds for >10% differences in methylation. **k**, Metastatic outgrowth calculated as fold change to day 1.5 injected control mice of control D2A1 cells and D2A1 cells that were treated with decitabine overnight, or, **l**, for 36 hrs. Data are presented as mean values +/− s.d., *p* values by two-tailed t-test are shown. n = 4–6 mice. **m**, Total tumor cell abundance in mice 14 days postinjection with 4T1 control cells or 4T1 cells that were treated overnight or for 36hrs with decitabine. Representative images show whole metastatic lungs of mice injected with control cells (left panel), overnight treated cells (mid panel) and cells treated for 36hrs (right panel). Data are presented as mean values +/− s.d., *p* values by one-way ANOVA with Tukey post-test are shown. n = 6 mice. **n**, Schematic of experiment. 4T1-GFP cells were treated overnight with either decitabine (Hypo Ctrl) or decitabine and SKL2001 (Hypo G-O-F) and injected into the tail veins of mice. Quantification shows the percentage of extravasated TC 1.5 days postinjection and, **o**, the percentage of TC with dye retention 3.5 days postinjection. Data are presented as mean values +/− s.d., *p* values were calculated by two-tailed t-test. n.s., not significant. n = 5 mice. **p**, Proliferation coefficients were calculated as the ratio of CellTrace mean fluorescence intensity (MFI) at day 3.5 and average CellTrace MFI at day 1.5 from matched experiments for 4T1-GFP control cells (Ctrl), SKL2001 reprogrammed cells (Ctrl G-O-F), over-night decitabine treated cells (Hypo Ctrl) and over-night decitabine and SKL2001 treated cells (Hypo G-O-F). Low proliferation coefficients reflect high proliferation rate. Cells with dye retention were excluded from the analysis. Data are presented as mean values +/− s.d., *p* values were calculated by one-way ANOVA with Tukey post-test. n.s., not significant. n = 5 mice. Source data

Because metastatic latency was established independently of differential exogenous factors such as ligand availability, receptor repertoire and niche occupancy, we probed for TC-intrinsic properties that could drive the observed differential Wnt responsiveness. Although growing in a Wnt-deficient environment, cultured TCs exhibited a heterogeneous but correlating baseline expression of EMT- and Wnt pathway-associated genes (Fig. 6a–c). Such differences in state were also identified in freshly isolated CTCs of patients with breast cancer^34,35^ (Extended Data Fig. 8a), indicating baseline TC-intrinsic differences. Moreover, overnight pulse treatment with Wnt agonists failed to program the extravasation–latency shift observed for long-term treatments (Fig. 6d and Extended Data Fig. 8b), and the expression profile of key EMT-associated transcription factors was not markedly changed between pulse-treated and reprogrammed cells (Extended Data Fig. 8c,d). This led us to hypothesize that TCs were restricted in their responsiveness towards niche-derived factors by an epigenetic barrier. In agreement with this, overnight pulse treatment of 4T1-GFP with a demethylating agent (decitabine) enabled TCs to respond to niche-derived factors and to preferably follow the extravasation–latency route (Fig. 6e). This was verified in a microscopy-based approach and, together with the finding that the viability of hypomethylated TCs was not affected and that decitabine treatment neither induced EMT in vitro nor altered the homing—but only the outgrowth—capacity of TCs (Extended Data Fig. 9a–e), indicated the initial priming of hypomethylated TCs for latency. Similar to the Wnt manipulation experiments, the short-term effects of decitabine treatment were indicative of the long-term outcome because hypomethylation resulted in a markedly reduced metastatic burden 2 weeks postinjection (Extended Data Fig. 9f). Moreover, the link between hypomethylation and latency induction appeared to be more general because decitabine treatment of the human breast cancer line MDA-MB-231 and melanoma cell line B16F10 led to an even stronger reduction in metastatic burden as compared with 4T1 cells (Extended Data Fig. 9g,h). To test whether the baseline methylation state of TCs correlates with their metastatic capacity, methylation array analysis on cancer cell lines of varying metastatic potential was performed. Remarkably, the most metastatic cell line (4T1) was also found to have the highest baseline methylation level whereas the dormant cell line (D2.0R) was the least methylated (Extended Data Fig. 9i,j). These data suggested a putative dose–effect of hypomethylation and, indeed, for both D2A1 and 4T1 cells an aggravated phenotype was observed by prolonging the overnight treatment of decitabine to 36 h (Extended Data Fig. 9k–m).

Importantly, latency induction of hypomethylated 4T1 cells was still dependent on Wnt because Wnt depletion led to a significant reduction in LTCs while simultaneously enhancing the short-term metastatic burden (Fig. 6f). Notably, pulse treatment of hypomethylated cells with Wnt agonist before injection did not alter the in vivo phenotype, indicating that niche-derived signaling was saturated and sufficient to induce latency (Extended Data Fig. 9n,o). Moreover, none of the in vitro treatments affected the proliferation rate of proliferation-committed 4T1 cells in vivo (Extended Data Fig. 9p), showing that differences in both short- and long-term metastatic burden were a consequence of latency induction.

**Extended Data Fig. 10:**
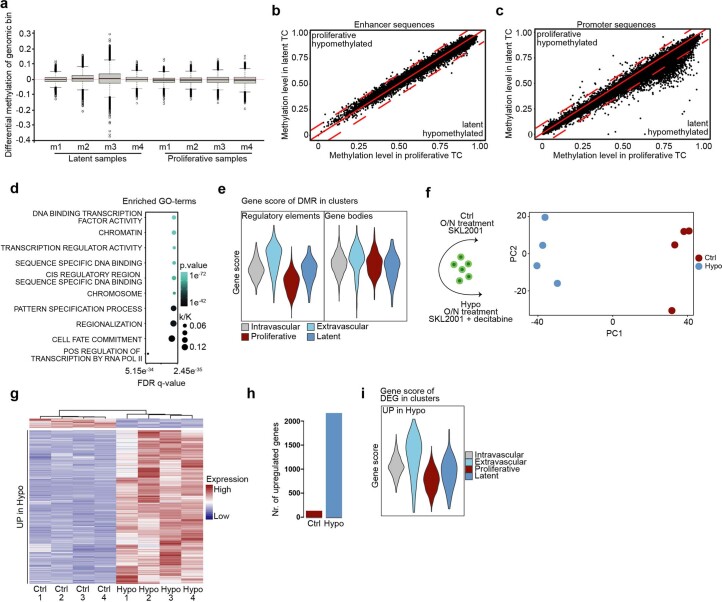
Latent tumor cell hypomethylation in promoter sequences and gene bodies drives plasticity. **a**, Boxplot reflecting the genome wide differential methylation level. Genome was binned in parts of 2000 bp. Methylation fractions were calculated for each bin by the ratio of methylated to total CpG island per bin. Methylation levels were assessed by subtracting the median methylation fraction of a bin across all samples from each individual bin. Red line indicates median methylation fraction of genomic bin. Black lines indicate median for each sample. Top and bottom of box show 25th and 75th percentile, respectively. Whiskers represent the 1.5 interquartile range. **b**, Scatter plot of methylation level (fraction of methylated CpG islands) for enhancer or **c**, promoters in latent TC and proliferative TC. Red line indicates no differences in methylation, dotted red lines indicate thresholds for >10% differences in methylation. **d**, Enriched GO-terms for hypomethylated genes in latent TC. k/K, overlap of queried gene list and GO-term associated genes. FDR, false discovery rate. *p* values were computed by permutation. **e**, Scored gene expression of genes linked to hypomethylated regulatory elements in latent tumor cells (as identified in panels **b**, **c**) (left panel) or gene bodies (Fig. 5g) (right panel) in the scRNAseq TC dataset split by cell clusters. **f**, Schematic of RNAseq experiment. Tumor cells were either treated with SKL2001 (Ctrl) or SKL2001 and decitabine (Hypo) overnight. PCA plot of sequencing results (right panel). n = 4 replicates. **g**, Row normalized and row clustered heatmap of differentially expressed genes between Ctrl and Hypo samples. Genes were considered significantly regulated for log 2 fold changes > 0.58 and adjusted *p* value < 0.05. *p* values were computed in DESeq2 using Wald test. **h**, Bar plot displaying number of upregulated genes for each treatment group (full set of DEG is displayed in Supplementary Table 9). **i**, Scored gene expression of Hypo gene signature from the RNAseq experiment in the scRNAseq dataset split by cell clusters.

We then assessed the methylation state of latent and proliferative TCs by performing whole-genome bisulfite sequencing on bulk-sorted TC fractions according to the gating strategy used for the scRNA-seq experiment. Overall methylation levels were not changed in LTCs, but promoter sequences and gene bodies showed considerable hypomethylation while enhancer sequences were mainly unaffected (Fig. 6g and Extended Data Fig. 10a–c). Remarkably, hypomethylation mainly occurred in genes and promoters that were epigenetically sealed in proliferative TCs (>70% methylation). This finding, together with the previous observation that TCs occupy distinct baseline Wnt and EMT states, support a model in which demethylation would occur stochastically in vitro before the injection of cells with niche factors instructing epigenetically susceptible TCs to acquire a latent phenotype, rather than a signaling-directed demethylation event with subsequent selection in vivo. In line with that, computing the overlap of genes with >10% hypomethylation in LTCs and Gene Ontology terms from the Molecular Signatures Database (MSigDB)^36^ revealed general biological programs such as transcription factor binding and cell fate processes as top hits (Extended Data Fig. 10d). Moreover, genes linked to differentially methylated regulatory elements in LTCs were enriched in extravascular cells, and in LTCs compared with proliferative TCs, whereas the expression of genes with hypomethylated gene bodies was not changed (Extended Data Fig. 10e). This indicated that hypomethylation of regulatory elements drives a gene expression program that enables TC extravasation and latency. To substantiate these findings, control and hypomethylated TCs were pulse treated with SKL2001 and RNA-seq was performed to assess TC responsiveness towards Wnt signaling (Extended Data Fig. 10f and Supplementary Table 9). As expected, hypomethylated TCs showed enhanced responsiveness towards canonical Wnt pathway activation and resembled extravascular and latent cells compared with proliferative cells at the transcriptional level (Extended Data Fig. 10g–i).

Collectively, these data confirm our hypothesis that hypomethylation underlies cellular plasticity and is the driving force of TC responsiveness towards niche-derived, latency-inducing factors.

## Discussion

Tumor cell predetermination is an emerging concept^37–39^. Here we identified the epigenetic precoding of disseminated TC behavior in the metastatic niche. Metastatic LTCs were characterized by hypomethylation in promoter sequences and gene bodies whereas proliferative TCs were epigenetically sealed. We envision that the plastic–latent and sealed–proliferative states form a dynamic equilibrium. Long-term treatment with Wnt agonist would direct the equilibrium towards the plastic–latent state without affecting the cell state itself. This is supported by reports of similar phenotype transitions resulting from long-term in vitro treatments or targeted genetic manipulation of signaling pathways^40,41^. However, such state transitions were also reported to occur spontaneously and were found to be a prerequisite for metastatic outgrowth^42^. TC hypomethylation was reflected at the transcriptomic level by an elevated baseline expression of EMT and Wnt pathway-associated genes. A similar heterogeneous expression was found in freshly isolated CTCs from patients with breast cancer and could be linked directly to their metastatic potential^34,43^. In this context, the primary tumor could be viewed as a heterogenous amplifier in which high selective pressure forces the acquisition of distinct TC states. Recent lineage-tracing experiments highlighted this phenomenon and revealed hybrid EMT TC states as the underlying principle of metastatic dissemination^6–9,39,44^. While EMT was needed for migration and intravasation, too much of it limited metastatic outgrowth. Interestingly, hybrid EMT states were not discrete but formed a continuum that correlated with the metastatic outcome^7–9,44^. We observed similar gradual states in cultured TCs, which could be a direct consequence of epigenetic plasticity. Plastic cells would show high EMT and follow the extravasation–latency route whereas epigenetically sealed TCs would form macrometastases. Probing the epigenetic and transcriptomic state of CTCs could therefore serve as a predictive tool to assess the likelihood of metastatic relapse in patients.

Besides cell-intrinsic properties, disseminated TC phenotypes are established as a consequence of instructive niche-derived factors. Here we identified endothelium-derived angiocrine Wnt signaling as a prototypic example of such dependency. However, other factors and other cellular sources have been identified previously and are most likely to act synergistically^10,20,21,45–49^. Most surprisingly, homeostatic angiocrine Wnt signaling was found to be sufficient to drive latency induction, suggesting a default tumor-suppressive lung niche. Similar default programs could occur in other organs in which ECs comprise a major Wnt source and were reported previously in different contexts^10,12^. Moreover, primary tumor-instructed remodeling of the niche could change the default state^50–54^. In addition, the data suggest that metastatic TCs actively inflicted a niche EC gene program that resembles primary tumor EC signatures^33,55^ and that could fuel TC proliferation through alterations of the biophysical properties of the microniche and the local production of biomass.

Collectively, these data provide an important insight into the establishment of metastasizing TC fates. We show that susceptible epigenetic states render TCs responsive towards niche-derived default factors, thus opening the opportunity to probe for TC–niche interdependencies at the systems level. However, a more focused effort is needed to investigate whether the susceptible epigenetic states observed in cultured TCs in this study are similarly present in primary tumors and transmitted with metastasizing TCs. Moreover, it remains elusive whether fixed epigenetic states are switchable at distant sites over time, especially in human cancers.

## Methods

### Animal studies

All animal work was performed in accordance with German national guidelines on animal welfare and the regulations of the regional council of Karlsruhe under permit nos. G-164/16, G-107/18, G-251/20, DKFZ305 and DKFZ370. Female NOD-SCID and BALB/c mice were acquired from Janvier Labs. B6 *Pdgfb-*iCreERT2*-*IRES*-EGFP* × *Wls* floxed mice were bred in the barrier animal facilities of the German Cancer Research Center. Mice were housed in sterile cages, maintained in a temperature-controlled room and fed autoclaved water and food ad libitum. All animals were monitored daily for signs of disease, and ear punches were used for genotyping the mice. Imported mice were allowed to acclimatize for a minimum of 7 days before each experiment. For all experiments, 8–12-week-old mice were used and euthanized via rapid cervical dislocation of the spinal cord at the experimental endpoint. For genetic experiments, female and male mice were used.

### Cell culture

Cell lines 4T1, 4T1-GFP, D2.0R, D2A1-tom and E0771-GFP were gifts from the laboratories of R. Weinberg (Whitehead Institute, Cambridge, MA), J. Sleeman (Heidelberg University, Mannheim, Germany) and K. Hodivala-Dilke (Barts Cancer Institute, London, UK). B16F10 and MDA-MB-231 cells were purchased from ATCC. All cells were maintained at 37 °C and 5% CO_2_ under high humidity and cultured in high-glucose DMEM (Gibco) with 10% (v/v) fetal calf serum (FCS) and 100 U ml^−1^ penicillin/streptomycin (Sigma-Aldrich). D2.0R-GFP, MDA-MB-231-GFP and 4T1-mCherry cells were generated by lentiviral transduction with TurboGFP and mCherry reporter, respectively. Niche-labeling cells were generated by lentiviral transduction of 4T1-GFP and D2.0R-GFP cells, as described previously^56^. In brief, cells were transduced with niche-labeling lentivirus provided by the laboratory of I. Malanchi (Francis Crick Institute, London, UK). Cells were purified by sorting mCherry^+^ cells to homogenous and a stable mCherry^+^ culture was established. Cells were checked regularly for mycoplasma contamination by PCR, and cell identity was confirmed by morphology. Cells were subcultured on reaching 80–90% confluency by treatment with trypsin-EDTA (Sigma-Aldrich). For in vitro treatments, cell media were supplemented with 20 mM SKL2001 in DMSO (Selleckchem), 3 mM in DMSO CHIR99021 (Selleckchem), 1 µM in PBS decitabine (Sigma-Aldrich) or 100 ng ml^−1^ in PBS human/mouse recombinant WNT5A (R&D). Cells were treated overnight (~17 h) for pulse treatment (SKL2001, CHIR99021 and decitabine), with 2 weeks for reprogramming with media changes either daily (SKL2001 and CHIR99021) or every 2 days (WNT5A), containing either drugs or vehicle (solvent only).

### Metastasis models and treatments

For experimental metastasis, TCs were resuspended in 200 µl of PBS and injected into the tail vein of mice. For transcriptomic and epigenomic screening experiments (Figs. 1a and 4a), female BALB/c mice were injected twice with 1 × 10^6^ TCs (4T1-GFP, niche-labeling 4T1-GFP, niche-labeling D2.0R-GFP) with a 30-min break between injections. For pharmacological treatment studies, female BALB/c mice were injected once with 1 × 10^6^ 4T1-GFP or D2A1-tom cells for short-term experiments, 5 × 10^5^ cells for the 1-week time point and 1 × 10^5^ cells for the 2-week time point. For hypomethylation studies using B16F10, 2 × 10^5^ cells were injected in female C57Bl/6 mice and, for experiments using MDA-MB-231-GFP, 5 × 10^5^ cells were injected in female NSG mice.

For genetic KO experiments, male and female B6 *Pdgfb*-iCreERT2-IRES-*EGFP* × *Wls* floxed mice were injected with 2 × 10^5^ E0771-GFP or B16F10, respectively. For staining of intravascular cells, 5 µg (FACS) or 30 µg (microscopy) of fluorescently labeled anti-H-2Kd antibody in 50 µl PBS was injected intravenously 2 min before euthanasia.

Pharmacological depletion of Wnt was achieved by daily oral gavage of 10 mg kg^−1^ body weight LGK974 resuspended in 0.5% methylcellulose (Sigma-Aldrich) and 0.5% Tween 80 (Sigma-Aldrich) in PBS^57^.

Endothelial cell-specific depletion of Wnt ligands was achieved using B6 *Pdgfb*-iCreERT2-IRES-*EGFP* × *Wls* floxed mice. Genetic recombination was initiated by intraperitoneal delivery of 2 mg of tamoxifen (Sigma-Aldrich) dissolved in 50 µl of corn oil with 5% ethanol. Both Cre^+^ and Cre^−^ littermates received five consecutive daily injections and were subjected to a 1-week washout period before the start of the experiment.

For modeling of spontaneous dissemination, 1 × 10^6^ 4T1-GFP cells in 100 µl of PBS were injected into the inguinal mammary fat pad of female NOD-SCID mice. NOD-SCID mice, rather than BALB/c, were used to avoid GFP immunogenicity. Tumor volumes were assessed by calliper measurement (tumor volume = 0.5 × length × width^2^). Once tumor sizes reached 100 mm³, mice were treated daily with LGK974 for 5 days with subsequent resection of the primary tumor as detailed above. LGK974 treatment continued for 2 days postresection and mice were left to develop metastases for 2 weeks.

To account for general inflammatory signatures in lung ECs in the niche-labeling experiment (Fig. 5b,c), mice were injected intraperitoneally with 1 mg kg^−1^ LPS (Sigma-Aldrich) in 0.9% NaCl (Braun) 24 h before euthanasia.

Lungs were collected in PBS and metastatic foci were counted (B16F10 experiments) and imaged using a stereomicroscope (Leica) (E0771 and primary tumor experiments) and processed for flow cytometry. Resected primary tumors were rinsed in PBS and fixed in formalin-free Zn buffer.

Mice that reached termination criteria before the experimental endpoint were excluded from the experiment. These criteria included overall bad state of health, weight loss and—for lung metastasis experiments—tachypnea. To adhere to the 3R principles (replacement, reduction and refinement), the data presented in Fig. 3e and Extended Data Fig. 9e utilized the same control animals. As such, all animals and cells were treated with either vehicle control or the respective pharmacological agent. Similarly, experiments shown in Extended Data Figs. 4h and 9c utilized the same controls.

### Isolation of lung cells

Lungs were minced on ice using curved, serrated scissors. The minced tissue was resuspended in DMEM supplemented with Liberase Thermolysin Medium enzyme mix (0.2 mg ml^−1^, Roche) and DNAse I (0.2 mg ml^−1^, Sigma-Aldrich) and incubated at 37 °C, first for 15 min and then again for 12 min. Following each incubation, minced tissues were passed through 18-G cannula syringes. Following the second incubation, digested tissues were passed through a 100-µm cell strainer. FCS was added and samples were centrifuged. Erythrocytes were lysed using prechilled 1× ammonium chloride potassium (ACK) buffer and the reaction was quenched by the addition of ice-cold PBS.

### Flow cytometry analysis and FACS sorting

Single-cell suspensions were passed through a 40-µm cell strainer and preincubated with antimouse CD16/CD32 Fc block (1:100, Thermo Fisher Scientific) for 15 min in flow buffer (PBS supplemented with 5% (v/v) FCS) and, subsequently, with the appropriate antibody mix (Supplementary Materials Table) for 20 min on ice.

Dead cells were excluded by staining with either FxCycle^TM^ Violet Stain (1:1,000, Thermo Fisher Scientific) or Fixable Viability Dye eFluor^TM^ 780 (1:1,000, Thermo Fisher Scientific) according to the manufacturer’s instructions. All samples were gated on viable cells followed by exclusion of cell doublets and CD45^+^, LYVE1^+^, PDPN^+^ and TER119^+^ cells using BD FACS Diva Software (BD Biosciences). For flow cytometry, samples were recorded on a BD LSR Fortessa or BD FACSCanto II cell analyzer (both BD Biosciences) and flow data were analyzed with FlowJo software (BD Biosciences, v.10). Tumor cell frequencies were calculated either as a percentage of sample-matched lung endothelial cells (TC number), as total TC counts per whole lung or normalized to milligrams of lung tissue using CountBright^TM^ Absolute Counting Beads according to the manufacturer’s protocol. Cells were sorted using a BD Biosciences Aria cell sorting platform with 100-µm nozzle.

#### Gating of extravascular TCs

A representative gating strategy for quantification of extravascular TCs is shown in Extended Data Fig. 4a. Lung endothelial cells were used as a positive control to set the intravascular gates. As such, one sample of the control group was used to set the gates in such a way that all ECs fell into the intravascular gate. This gate was then pasted to all samples and gate quality was assessed by checking the EC gate for each individual sample. Because H-2Kd staining is an in vivo method that might be subjected to greater variability, gates were adjusted if marked differences were observed. Samples for which cells were not sufficiently stained were excluded from the experiment. The adjusted gates were then pasted to the TCs and extravascular cells quantified.

#### Gating of LTCs

A representative gating strategy for quantification of LTCs is shown in Extended Data Fig. 4b,c. For each experiment, one additional mouse was used that served as latent control and which was euthanized at day 1.5 to set the gate for nonproliferative TCs. Because in vitro treated cells (Wnt G-O-F and hypomethylation experiments) were stained with CellTrace in separate staining mixes, a control for each treatment was included (Extended Data Fig. 4b) to account for staining batch effects. Gating was performed for each treatment group in a way that all day 1.5 TCs fell into the latent gate. Day 1.5 gates were then pasted to all samples from the respective day 3.5 treatment group and LTCs quantified.

### scRNA-seq

scRNA-seq was performed using a modified SMART-Seq2 protocol^58^. In brief, single cells were sorted directly into 96-well plates containing 1 µl of lysis buffer per well, centrifuged and snap-frozen in liquid nitrogen. For CD45^−^ PDPN^−^ LYVE^−^ TER119^−^ CD31^+^ ECs, four plates (384 cells) were sorted for three biological replicates from days 1.5 and 3.5 (total of 1,152 cells per time point). Day 0 control samples were split and three plates (288 cells) were sorted for two biological replicates on days 1.5 and 3.5 (total of 1,152 cells), respectively, to account for technical batch effects. For TCs, one plate of matched intra- and extravascular fractions (each 96 cells) was sorted from four biological replicates (total of 384 cells per fraction) on day 1.5. Similarly, one plate of matched latent and proliferative fractions was sorted from four biological replicates on day 3.5. Frozen plates were thawed on ice and oligo-dT-primer (see Supplementary Materials Table for a detailed list of primers used) were annealed at 70 °C for 3 min. Next, 1.3 µl of reverse transcription mix with template-switching oligo was added to each well and isolated mRNA was transcribed to full-length complementary DNA. Full-length cDNA was amplified by the addition of 2.4 µl of PCR mastermix to each well. EC and TC cDNA were amplified using 22 and 18 cycles, respectively. Amplified cDNA was purified using AMPure XP beads (Beckman Coulter), and random wells were selected for quality control using a 2100 Bioanalyzer (Agilent) and a Qubit fluorometer (Thermo Fisher Scientific). DNA concentration for each well was measured using the Quant-iT^TM^ high-sensitivity kit (Thermo Fisher Scientific) and concentrations were manually adjusted to 0.1–0.3 ng µl^−1^. Tagmentation was performed using the Nextera XT DNA library preparation kit (Illumina) and a mosquito liquid handler (SPT Labtech), with the addition of 1.2 µl of Nextera XT-TD buffer mix to 0.4 µl of cDNA. ECs were pooled according to biological replicate whereas TC replicates were pooled according to sort gate. Customized i5 and i7 index primers were added and tagmented cDNA was amplified using 14 PCR cycles. Wells from each plate were pooled and multiplexed libraries purified and quality controlled using TapeStation (Agilent) and a Qubit fluorometer (Thermo Fisher Scientific). Multiplexes were sequenced on individual lanes on a HiSeq2000 (Illumina) using a V4 50-cycle single-read kit generating ~500.000 reads per cell.

### Bulk RNA-seq of labeled niche ECs

A total of 50,000 unlabeled lung ECs and sample-matched labeled ECs were sorted into RNase-free 1.5-ml microcentrifuge tubes containing 100 µl of lysis buffer and snap-frozen on dry ice. For each condition, six biological replicates were included. Snap-frozen RNA was extracted using an Arcturus PicoPure RNA Isolation Kit (Thermo Fisher Scientific) according to the manufacturerʼs instructions. RNA was quality controlled using a Qubit fluorometer (Thermo Fisher Scientific) and 2100 Bioanalyzer (Agilent). Samples with RNA integrity number <8 were discarded. RNA was transcribed to full-length cDNA using the SMART-Seq2 (ref. ^58^) protocol and RNA-seq libraries were generated using the NEBNext Ultra^TM^ II FS DNA library preparation kit (New England Biolabs), according to the manufacturer’s protocol, with DNA input <100 ng. Libraries were pooled into one multiplex and sequenced over two lanes on a NovaSeq 6000 using the S1 100-cycle paired-end kit generating ~35 × 10^6^ reads per sample.

### Bulk RNA-seq of cultured TCs

Cultured TCs were treated overnight with either 20 mM SKL2001 (control samples) or 20 mM SKL2001 and 1 µM decitabine (hypomethylation samples). Cells were lysed and RNA extracted using the GenElute Total RNA Purification Kit (Sigma-Aldrich) according to the manufacturer’s instructions. RNA integrity was assessed by TapeStation (Agilent), and samples with RNA integrity number <9 were excluded. Sequencing libraries were prepared using the Illumina TruSeq mRNA stranded Kit following the manufacturer’s instructions. Briefly, mRNA was purified from 500 ng of total RNA using oligo(dT) beads. Next, poly(A)^+^ RNA was fragmented to 150 base pairs (bp) and converted to cDNA. cDNA fragments were end repaired, adenylated on the 3′ end, adapter ligated and amplified with 15 cycles of PCR. Final libraries were validated using Qubit (Invitrogen) and TapeStation (Agilent Technologies). Finally, 2 × 100-bp paired-end sequencing was performed on an Illumina NovaSeq 6000 according to the manufacturer’s protocol. At least 54 x 10^6^ reads per sample were generated.

### Whole-genome bisulfite sequencing

Lungs from six mice were pooled into one sample and 200.000 proliferative TCs and total LTCs were sorted from four pools and snap-frozen on dry ice. Genomic DNA was extracted using the NucleoSpin tissue minikit for DNA from cells and tissue (Macherey-Nagel). DNA integrity was assessed using TapeStation (Agilent) and samples with DNA integrity number <7 were discarded. Whole-genome bisulfite sequencing libraries were prepared using the xGen^TM^ Methyl-Seq DNA Library Prep Kit (IDT) with partially modified steps in bead clean-up/size selection. Briefly, 200 ng of gDNA was fragmented to 700–1,000 bp using a Covaris ultrasonicator and quality checked with TapeStation (Agilent Technologies). Fragmented DNA samples were treated with bisulfite using the EpiTect Bisulfite Kit (Qiagen) following the Illumina instructions (part no. 15021861 rev. B). Adapters were attached to the 3' ends of single-stranded DNA fragments and extended. Double-stranded DNA fragments were cleaned up using 1.6× AMPure XP beads (Beckman Coulter) and size selected with a bead ratio of 0.6x and 0.2x, followed by ligation of truncated adapter 2 to the uracil-free strand. The adapter-ligated libraries were enriched and indexed using six cycles of PCR and purified by magnetic beads according to the protocol provided. Amplified libraries were quality checked using a Qubit fluorometer (Thermo Fisher Scientific) and TapeStation (Agilent). Libraries were pooled equimolarly into one multiplex and sequenced over two lanes on a NovaSeq 6000 using the S1 150-cycle, paired-end kit, enabling an average genomic coverage of >15.

### Methylation array of cultured TC lines

Cells were lysed in the dish and DNA was extracted using the NucleoSpin tissue minikit for DNA from cells and tissue (Macherey-Nagel). Genome-wide screening of DNA methylation patterns was performed using Infinium MouseMethylation285k BeadChips (Illumina). DNA concentrations were determined using PicoGreen (Molecular Probes, Inc.), quality controlled by agarose-gel analysis and samples of average fragment size >3 kb were selected for methylation analysis. First, 500 ng of gDNA from each sample was bisulfite converted using the EZ-96 DNA Methylation Kit (Zymo Research Corporation) according to the manufacturer’s recommendations. Each sample was whole-genome amplified and enzymatically fragmented following the instructions provided in the Illumina Infinium HD Assay Methylation Protocol Guide. DNA was applied to Infinium MouseMethylation285k BeadChips and hybridization performed for 16–24 h at 48 °C. Allele-specific primer annealing was followed by single-base extension using DNP- and biotin-labeled dideoxy-nucleoside triphosphates. Following extension the array was fluorescently stained and scanned and intensity at each CpG measured. Microarray scanning was done using an iScan array scanner (Illumina).

### Niche-labeling RNA-seq analysis

Raw sequencing data were demultiplexed and FASTQ files generated using bcl2fastq software (Illumina, v.2.20.0.422). FASTQ files were mapped to the GRCm38 mouse reference genome using salmon (v.0.7.2)^59^, and count matrices were constructed with the R package tximport (v.1.18.0)^60^. Differential gene expression analysis was performed using DESeq2 (v.1.30.1)^61^. Each condition was tested against each condition and differentially expressed genes were used for GSEA^62,63^, which was performed using either the R package clusterProfiler (v.3.18.1)^64^ or the GSEA java desktop application and MSigDB (v.7.4)^36^ provided by the Broad Institute.

For the proliferative niche EC gene panel, differential gene expression analysis was performed between all groups (each group against each group). Genes were considered significantly upregulated at *P* < 0.01 and log_2_ fold change (FC) > 0.5. Significantly regulated genes were considered marker genes of a sample type if the gene was specifically upregulated for one sample type (for example, upregulated only in proliferative niche ECs across all comparisons) and also if that gene was upregulated for at least three out of the five comparisons. A full set of all regulated genes and putative marker genes is presented in Supplementary Tables 6 and 7.

For deconvolution of bulk samples, aCap and gCap marker genes were defined using the scRNA-seq dataset (Supplementary Table 1). Expression coefficients (aCap/gCap) of summed marker genes were calculated for each bulk sample using quasibinomal fitting and normalized to PBS-injected control samples. Resulting ratios were exponentiated for plotting.

### Analysis of RNA-seq of cultured TCs

Reads were subjected to quality trimming with the following criteria: (1) minimal Phred score of Q15 (default), (2) fraction of unqualified bases allowed in a single read was set to 40% (default) and (3) minimal length of read to be included was set to 15 bp. No adapter trimming was performed. Trimmed reads were aligned against the mm10 reference genome with the GRCm38.96 transcript annotation file using STAR^65^ in htseq-count^66^ mode to obtain gene-level read counts. Differential gene expression analysis and data normalization were performed using DESeq2 (v.1.30.1)^61^. A full set of differentially expressed genes is presented in Supplementary Table 8.

### scRNA-seq analysis

#### Preprocessing and normalization

Raw sequencing data were processed as described above. Gene expression was normalized to the mean expression of a housekeeping gene panel (*Actb*, *Gapdh*, *Tubb5*, *Ppia*, *Ywhaz*, *B2m*, *Pgk1*, *Tbo*, *Arbp*, *Gusb* and *Hprt1*) for each cell, scaled by a factor of 10.000 and log_10_ normalized. Normalized count matrices were analyzed using the R package Seurat (v.4.0.1)^67,68^. Gene and read counts per cell and percentage of mitochondrial transcripts were computed using the respective functions of the Seurat package. For the EC dataset, cells with a percentage of mitochondrial transcripts >5% and/or <1,000 genes were excluded. For the TC dataset, only those cells with a mitochondrial transcript percentage <5% and >2,500 genes were retained for further analysis.

#### Dimension reduction and clustering/classification

Shared nearest-neighbor (SNN)-based clustering and UMAP visualization were carried out using the FindClusters and RunUMAP functions on the basis of principal component analysis, which was performed using the RunPCA function. For TCs, dimensional reduction was performed on 15 principal components with resolution parameter set to 0.5 for clustering, whereas for ECs ten principal components were used. Clusters were annotated according to enrichment for cells derived from a specific FACS gate. For the EC dataset, cells were classified based on the scored expression of known lung EC subtype genesets (Supplementary Table 1). ECs with large-vessel gene score >1 were considered large-vessel ECs; ECs with cycle gene score >0.5 were considered as cycling ECs; ECs with aCap gene score >0 and gCap gene score <0 were considered as aerocytes; and general capillary ECs were defined as ECs with aCap gene scores <0 and gCap gene scores >−0.5. Contaminating cells were removed from the dataset based on the expression of either immune marker genes (*Ptprc*, *Itgam*, *Itgax*, *Adgre1*, *Cd3e*, *Cd19* and *Cd56*) or stromal cell and vessel mural cell marker genes (*Pdgfrb*, *Des*, *Myh11*, *Col1a2*, *Pdgfra*, *Cspg4*, *Pdpn* and *Acta2*).

#### Gene expression scoring and cell cycle analysis

Gene expression scoring was performed using the AddModuleScore function in Seurat. EMT, and Wnt gene sets (Supplementary Table 1) were compiled from MSigDB.

Cell cycle state was assessed by scoring cells for the expression of 43 S-phase-specific and 54 G2- or M-phase-specific genes^69^. Cells that originated from the latent FACS gate and had summed scores <−1 were tested against cells from the proliferative gate with score sums >0.

#### Trajectory analysis

Trajectory analysis of lung-resident TCs was performed using the R package Monocle (v.3 alpha)^70,71^. Clustering and dimension reduction were performed using default parameters in Monocle3. The trajectory graph was built by setting cells from the intravascular sorting gate as the starting point. Cells were colored according to cluster identities as identified in Seurat. Gene expression of EMT and Wnt gene sets was visualized using the plot_cells function. EMT and Wnt gene set enrichment along the trajectories was assessed by manual selection of cells on the respective trajectory graph using the choose_cells() function in Monocle3. The Seurat object was then subset based on cell IDs to obtain Seurat objects with cells located on the respective branches of the trajectory. Pseudobulks reflecting biological replicates were formed and the mean gene score for each replicate was calculated. The trajectory shown in Extended Data Fig. 2i was computed using the Seuratwrapper for Monocle.

#### Analysis of TC–EC interactions

Tumor cell pseudobulks reflecting a biological replicate and sort gate were formed, and DEGs were computed using DESeq2 (ref. ^61^). DEGs were filtered against the CellPhoneDB^31^ database to retrieve putative ligands and receptors. These were then filtered for the expression of interaction partners in either the day 1.5 EC dataset (for intravascular versus extravascular comparison) or the day 3.5 EC dataset (for latent versus proliferative comparison). log_2_ FCs of TC-expressed ligands or receptors were plotted against each other. Receptors or ligands with upregulation in extravasated and LTCs were considered trajectory defining, as well as those upregulated in intravascular and proliferative TCs.

#### Analysis of publicly available human CTC data

Normalized and filtered count matrices were downloaded from either the source data provided^34^ or the Gene Expression Omnibus under accession code GSE109761 (ref. ^35^). Dimension reduction and visualization were performed in Seurat as described above using default parameters. Gene scores of human orthologs of the EMT and Wnt gene lists were computed as described above.

### Whole-genome bisulfite sequencing data analysis

Raw sequencing data were demultiplexed and FASTQ files generated using bcl2fastq software (Illumina, v.2.20.0.422). FASTQ files were trimmed using Trimgalore (v.0.6.6)^72^ and mapped to the GRCm39 mouse reference genome using Bismark (v.0.22.3)^73^. Forward and reverse strands were collapsed and methylation sites were called in Bismark. Differentially methylated regions were determined with the R package bsseq using default parameters (v.1.26.0)^74^. Biological replicates were summed and methylation fractions for annotated genomic regions between the two conditions were compared. Regulatory elements, promoters and gene bodies were annotated with annotation sheets downloaded from the Ensembl database (release 105)^75^.

### Methylation array data analysis

Raw data obtained from the red and green channels were quantile normalized separately. The methylation indicator of a given target was assessed using GenomeStudio Methylation Module v.1.8 and beta-values were calculated as

$${\rm{beta}}=({\rm{grn}}.{\rm{B}})/({\rm{grn}}.{\rm{B}}+{\rm{grn}}.{\rm{A}}+100)$$

$${\rm{beta}}=({\rm{red}}.{\rm{B}})/({\rm{red}}.{\rm{B}}+{\rm{red}}.{\rm{A}}+100).$$

Each target on the chip was measured by two probe IDs (A and B), which were considered independent variables and for which separate beta-values (grn.A/B or red.A/B) were calculated. Aggregation of beads and calculation of mean values over groups of samples was done for each variable separately. Means, standard deviations and *P* values of grn.A/B and red.A/B were calculated and Benjamini–Hochberg correction was performed. For differential methylation analysis the difference in beta-values was calculated using the mean beta-values of the two groups, which were then compared.

The statistical significance of each difference was assessed by calculation of the two *P* values for each beta-value on the bead level and choosing the minimum of both. In addition, *t*-test *P* values were calculated using the beta-values for each sample in the group. For global statements about overall methylation effects, *P* values were adjusted using Benjamini–Hochberg correction. In addition, *M*-values were calculated from beta-values (raw and normalized) as follows:

$${\rm{mvalue}}.{\rm{grn}}={\log}_{2}(({\rm{beta}}.{\rm{grn}}.{\rm{B}}+1)/({\rm{beta}}.{\rm{grn}}.{\rm{A}}+1))$$

$${\rm{mvalue}}.{\rm{red}}={\log}_{2}(({\rm{beta}}.{\rm{red}}.{\rm{B}}+1)/({\rm{beta}}.{\rm{red}}.{\rm{A}}+1)).$$

### Real-time quantitative PCR

Total RNA of cell-cultured TCs was isolated using the GenElute Mammalian Total RNA Purification Kit (Merck) according to the manufacturer’s instructions. First, 1,000 ng of RNA was reverse transcribed using the QuantiTect Reverse Transcription Kit (Qiagen) according to the manufacturer’s protocol. Gene expression analysis was performed by quantitative PCR using TaqMan reactions (Thermo Fisher Scientific) (Supplementary Materials Table) and Lightcycler 480 (Roche). Gene expression levels were assessed using the Ct method and normalized to the expression of Actb, resulting in ΔCt values. Relative gene expression was assessed by normalization of ΔCt values of individual samples to the average control ΔCt value, resulting in ΔΔCt values. Relative FCs to control were then calculated as 2^−^^ΔΔCt^.

### Histology

Zinc-fixed primary tumors were paraffin embedded and cut into 7-µm sections. These were deparaffinized and rehydrated and antigen retrieval was performed by incubation with Proteinase K (20 µg ml^−1^, Gerbu Biotechnik) for 5 min at 37 °C. Tissues were blocked in 10% ready‐to‐use goat serum (Zymed) for 1 h at room temperature, followed by overnight incubation with rat anti-CD31 (1:100, BD Biosciences) and rabbit anti-Desmin (1:100, abcam) in blocking buffer at 4 °C. Following three washes in Tris buffered saline with Tween, slides were stained with antirat Alexa 647 and antirabbit Alexa 546 antibody at room temperature for 1 h. Cell nuclei were counterstained with 1:2,000 Hoechst 33342 (Sigma-Aldrich) and sections mounted with DAKO mounting medium (Agilent). Images were acquired as whole-area tile scans using an Axio Scan.Z1 slide scanner (Zeiss). Image analysis was performed using Fiji software (ImageJ, 1.53q). Following region-of-interest selection, CD31, Desmin and DAPI channels were binarized using thresholding. For vessel area, the percentage of CD31^+^ area within the region of interest was calculated. For vessel coverage the CD31 channel was masked and Desmin overlap with CD31 was calculated. CD31^+^/Desmin^+^ double-positive vessels were considered covered, and coverage was calculated as the ratio of covered:total vessels.

Cryo-embedded lung tissues were cut into sections of either 12 µm (experiments for counting single disseminated tumor cells) or 30 µm (anti-H-2Kd staining experiments). Sections were blocked and permeabilized in 0.3% Triton X-100 and 10% FCS in PBS for 1 h at room temperature, followed by overnight incubation with goat anti-CD31 (1:100, R&D) in blocking/permeabilization buffer at 4 °C. Sections were washed three times with 0.2% Triton X-100 and 5% FCS in PBS, followed by overnight incubation with antigoat Alexa 568 antibody at 4 °C in washing buffer. Cell nuclei were counterstained with 1:2,000 Hoechst 33342 (Sigma-Aldrich) and sections mounted following three washes in DAKO mounting medium (Agilent). Representative images were acquired as z-stacks using either a Leica Sp8 or Leica Sp5 confocal microscope. Image analysis was performed in Fiji (ImageJ, 1.53q) for anti-H-2Kd experiments, and single disseminated tumor cells and cell clusters were counted manually under a fluorescence microscope (Zeiss).

### Statistical analysis

All statistical details of experiments (statistical test, *n* size, exact *P* values where applicable) can be found in the figure panels and respective legends. Data distribution was assumed to be normal but this was not formally tested. All statistical analyses were performed using either GraphPad Prism (v.6) or R (v.4.0.5). Sample sizes were chosen according to experience in the laboratory. For spontaneous metastasis models and experiments involving genetic recombination, sample sizes were predetermined using power analysis with the assumption of 75% proportional standard deviation and 60% difference in metastatic burden. Mice were randomly allocated to the experimental groups. Data collection and analysis were, wherever applicable, performed blinded.

### Reporting summary

Further information on research design is available in the Nature Portfolio Reporting Summary linked to this article.

### Supplementary information

Reporting Summary

Supplementary Tables Supplementary Tables 1–9 and one table containing detailed information regarding materials used in this study.

### Source data

Source Data Figs. 1, 3, 6 and Extended Data Figs. 1, 2, 4, 5, 6, 8, and 9 Statistical Source Data.

## Data Availability

All raw sequencing data and annotated and filtered count matrices were deposited in Gene Expression Omnibus under accession no. GSE221202. Methylation array data were deposited in ArrayExpress and can be accessed under accession no. E-MTAB-13432. Source data are provided with this paper. All other data supporting the findings of this study are available from the corresponding author on reasonable request.

## Code availability

All custom code generated in this study can be accessed via figshare with the following link: https://figshare.com/s/0a88bd5d86238bd3e1b3.

## References

[1] Risson E, Nobre AR, Maguer-Satta V, Aguirre-Ghiso JA (2020). The current paradigm and challenges ahead for the dormancy of disseminated tumor cells. Nat. Cancer.

[2] Polzer B, Klein CA (2013). Metastasis awakening: the challenges of targeting minimal residual cancer. Nat. Med..

[3] Labelle M, Hynes RO (2012). The initial hours of metastasis: the importance of cooperative host–tumor cell interactions during hematogenous dissemination. Cancer Discov..

[4] Ghajar CM (2015). Metastasis prevention by targeting the dormant niche. Nat. Rev. Cancer.

[5] Hüsemann Y (2008). Systemic spread is an early step in breast cancer. Cancer Cell.

[6] Fluegen G (2017). Phenotypic heterogeneity of disseminated tumour cells is preset by primary tumour hypoxic microenvironments. Nat. Cell Biol..

[7] Simeonov KP (2021). Single-cell lineage tracing of metastatic cancer reveals selection of hybrid EMT states. Cancer Cell.

[8] Lüönd F (2021). Distinct contributions of partial and full EMT to breast cancer malignancy. Dev. Cell.

[9] Yang D (2022). Lineage tracing reveals the phylodynamics, plasticity, and paths of tumor evolution. Cell.

[10] Ghajar CM (2013). The perivascular niche regulates breast tumour dormancy. Nat. Cell Biol..

[11] Baumann Z, Auf der Maur P, Bentires-Alj M (2022). Feed-forward loops between metastatic cancer cells and their microenvironment-the stage of escalation. EMBO Mol. Med..

[12] Crist SB (2022). Unchecked oxidative stress in skeletal muscle prevents outgrowth of disseminated tumour cells. Nat. Cell Biol..

[13] Barkan D (2010). Metastatic growth from dormant cells induced by a col-I-enriched fibrotic environment. Cancer Res..

[14] Albrengues, J. et al. Neutrophil extracellular traps produced during inflammation awaken dormant cancer cells in mice. *Science* **361**, eaao4227 (2018). 10.1126/science.aao4227 PMC6777850 30262472

[15] Dai J (2022). Astrocytic laminin-211 drives disseminated breast tumor cell dormancy in brain. Nat. Cancer.

[16] Strilic B, Offermanns S (2017). Intravascular survival and extravasation of tumor cells. Cancer Cell.

[17] Al-Mehdi AB (2000). Intravascular origin of metastasis from the proliferation of endothelium-attached tumor cells: a new model for metastasis. Nat. Med..

[18] Wong CW (2002). Intravascular location of breast cancer cells after spontaneous metastasis to the lung. Am. J. Pathol..

[19] Roncato, F. et al. Reduced lamin A/C does not facilitate cancer cell transendothelial migration but compromises lung metastasis. *Cancers*10.3390/cancers13102383 (2021). 10.3390/cancers13102383 PMC8157058 34069191

[20] Bragado P (2013). TGF-β2 dictates disseminated tumour cell fate in target organs through TGF-β-RIII and p38α/β signalling. Nat. Cell Biol..

[21] Nobre AR (2021). Bone marrow NG2(^+^)/Nestin(^+^) mesenchymal stem cells drive DTC dormancy via TGFβ2. Nat. Cancer.

[22] Niehrs C (2012). The complex world of WNT receptor signalling. Nat. Rev. Mol. Cell Biol..

[23] Yu M (2012). RNA sequencing of pancreatic circulating tumour cells implicates WNT signalling in metastasis. Nature.

[24] Miyamoto DT (2015). RNA-Seq of single prostate CTCs implicates noncanonical Wnt signaling in antiandrogen resistance. Science.

[25] Malladi S (2016). Metastatic latency and immune evasion through autocrine inhibition of WNT. Cell.

[26] Tammela T (2017). A Wnt-producing niche drives proliferative potential and progression in lung adenocarcinoma. Nature.

[27] Vila Ellis L (2020). Epithelial Vegfa specifies a distinct endothelial population in the mouse lung. Dev. Cell.

[28] Gillich A (2020). Capillary cell-type specialisation in the alveolus. Nature.

[29] Singhal M (2021). Temporal multi-omics identifies LRG1 as a vascular niche instructor of metastasis. Sci. Trans. Med..

[30] Hongu T (2022). Perivascular tenascin C triggers sequential activation of macrophages and endothelial cells to generate a pro-metastatic vascular niche in the lungs. Nat. Cancer.

[31] Efremova M, Vento-Tormo M, Teichmann SA, Vento-Tormo R (2020). CellPhoneDB: inferring cell–cell communication from combined expression of multi-subunit ligand–receptor complexes. Nat. Protoc..

[32] Ombrato L (2019). Metastatic-niche labelling reveals parenchymal cells with stem features. Nature.

[33] Goveia J (2020). An integrated gene expression landscape profiling approach to identify lung tumor endothelial cell heterogeneity and angiogenic candidates. Cancer Cell.

[34] Cheng YH (2019). Hydro-Seq enables contamination-free high-throughput single-cell RNA-sequencing for circulating tumor cells. Nat. Commun..

[35] Szczerba BM (2019). Neutrophils escort circulating tumour cells to enable cell cycle progression. Nature.

[36] Liberzon A (2011). Molecular signatures database (MSigDB) 3.0. Bioinformatics.

[37] Shaffer SM (2017). Rare cell variability and drug-induced reprogramming as a mode of cancer drug resistance. Nature.

[38] Emert BL (2021). Variability within rare cell states enables multiple paths toward drug resistance. Nat. Biotechnol..

[39] Quinn, J. J. et al. Single-cell lineages reveal the rates, routes, and drivers of metastasis in cancer xenografts. *Science* **371**, eabc1944 (2021). 10.1126/science.abc1944 PMC7983364 33479121

[40] Pattabiraman DR (2016). Activation of PKA leads to mesenchymal-to-epithelial transition and loss of tumor-initiating ability. Science.

[41] Zhang Y (2022). Genome-wide CRISPR screen identifies PRC2 and KMT2D-COMPASS as regulators of distinct EMT trajectories that contribute differentially to metastasis. Nat. Cell Biol..

[42] Fumagalli A (2020). Plasticity of Lgr5-negative cancer cells drives metastasis in colorectal cancer. Cell Stem Cell.

[43] Gkountela S (2019). Circulating tumor cell clustering shapes DNA methylation to enable metastasis seeding. Cell.

[44] Pastushenko I (2018). Identification of the tumour transition states occurring during EMT. Nature.

[45] Werner-Klein M (2020). Interleukin-6 trans-signaling is a candidate mechanism to drive progression of human DCCs during clinical latency. Nat. Commun..

[46] Ren D (2019). Wnt5a induces and maintains prostate cancer cells dormancy in bone. J. Exp. Med..

[47] Montagner M (2020). Crosstalk with lung epithelial cells regulates Sfrp2-mediated latency in breast cancer dissemination. Nat. Cell Biol..

[48] Pein M (2020). Metastasis-initiating cells induce and exploit a fibroblast niche to fuel malignant colonisation of the lungs. Nat. Commun..

[49] Correia AL (2021). Hepatic stellate cells suppress NK cell-sustained breast cancer dormancy. Nature.

[50] Peinado H (2017). Pre-metastatic niches: organ-specific homes for metastases. Nat. Rev. Cancer.

[51] Borriello L (2022). Primary tumor associated macrophages activate programs of invasion and dormancy in disseminating tumor cells. Nat. Commun..

[52] Costa-Silva B (2015). Pancreatic cancer exosomes initiate pre-metastatic niche formation in the liver. Nat. Cell Biol..

[53] Di Martino JS (2022). A tumor-derived type III collagen-rich ECM niche regulates tumor cell dormancy. Nat. Cancer.

[54] Liu Y, Cao X (2016). Characteristics and significance of the pre-metastatic niche. Cancer Cell.

[55] Lambrechts D (2018). Phenotype molding of stromal cells in the lung tumor microenvironment. Nat. Med..

[56] Ombrato L (2021). Generation of neighbor-labeling cells to study intercellular interactions in vivo. Nat. Protoc..

[57] Liu J (2013). Targeting Wnt-driven cancer through the inhibition of Porcupine by LGK974. Proc. Natl Acad. Sci. USA.

[58] Picelli S (2014). Full-length RNA-seq from single cells using Smart-seq2. Nat. Protoc..

[59] Patro R, Duggal G, Love MI, Irizarry RA, Kingsford C (2017). Salmon provides fast and bias-aware quantification of transcript expression. Nat. Methods.

[60] Soneson C, Love MI, Robinson MD (2015). Differential analyses for RNA-seq: transcript-level estimates improve gene-level inferences. F1000Res..

[61] Love MI, Huber W, Anders S (2014). Moderated estimation of fold change and dispersion for RNA-seq data with DESeq2. Genome Biol..

[62] Mootha VK (2003). PGC-1alpha-responsive genes involved in oxidative phosphorylation are coordinately downregulated in human diabetes. Nat. Genet..

[63] Subramanian A (2005). Gene set enrichment analysis: a knowledge-based approach for interpreting genome-wide expression profiles. Proc. Natl Acad. Sci. USA.

[64] Yu G, Wang LG, Han Y, He QY (2012). clusterProfiler: an R package for comparing biological themes among gene clusters. OMICS.

[65] Dobin A (2013). STAR: ultrafast universal RNA-seq aligner. Bioinformatics.

[66] Anders S, Pyl PT, Huber W (2015). HTSeq–a Python framework to work with high-throughput sequencing data. Bioinformatics.

[67] Stuart T (2019). Comprehensive integration of single-cell data. Cell.

[68] Hao Y (2021). Integrated analysis of multimodal single-cell data. Cell.

[69] Tirosh I (2016). Dissecting the multicellular ecosystem of metastatic melanoma by single-cell RNA-seq. Science.

[70] Trapnell C (2014). The dynamics and regulators of cell fate decisions are revealed by pseudotemporal ordering of single cells. Nat. Biotech..

[71] Qiu X (2017). Reversed graph embedding resolves complex single-cell trajectories. Nat. Methods.

[72] Krueger F. Trimgalore. *GitHub* https://github.com/FelixKrueger/TrimGalore (2021).

[73] Krueger F, Andrews SR (2011). Bismark: a flexible aligner and methylation caller for Bisulfite-Seq applications. Bioinformatics.

[74] Hansen KD, Langmead B, Irizarry RA (2012). BSmooth: from whole genome bisulfite sequencing reads to differentially methylated regions. Genome Biol..

[75] Cunningham F (2021). Ensembl 2022. Nucleic Acids Res..

