# Supplementary material for: Lung endothelium exploits susceptible tumor cell states to instruct metastatic latency
Source: Nat Cancer. 2024 Feb 2;5(5):716–30. doi: 10.1038/s43018-023-00716-7 (PMC11136671; doi:10.1038/s43018-023-00716-7)
Supplement: Supplementary file 1 — Reporting Summary [file 43018_2023_716_MOESM1_ESM.pdf]

nature portfolio

Corresponding author(s): Hellmut G Augustin
Moritz Jakab

Last updated by author(s): Nov 24, 2023

Reporting Summary

Nature Portfolio wishes to improve the reproducibility of the work that we publish. This form provides structure for consistency and transparency in reporting. For further information on Nature Portfolio policies, see our Editorial Policies and the Editorial Policy Checklist.

Statistics

For all statistical analyses, confirm that the following items are present in the figure legend, table legend, main text, or Methods section.

n/a	Confirmed	
☐	☒	The exact sample size (*n*) for each experimental group/condition, given as a discrete number and unit of measurement
☐	☒	A statement on whether measurements were taken from distinct samples or whether the same sample was measured repeatedly
☐	☒	The statistical test(s) used AND whether they are one- or two-sided<br>*Only common tests should be described solely by name; describe more complex techniques in the Methods section.*
☐	☒	A description of all covariates tested
☐	☒	A description of any assumptions or corrections, such as tests of normality and adjustment for multiple comparisons
☐	☒	A full description of the statistical parameters including central tendency (e.g. means) or other basic estimates (e.g. regression coefficient) AND variation (e.g. standard deviation) or associated estimates of uncertainty (e.g. confidence intervals)
☐	☒	For null hypothesis testing, the test statistic (e.g. *F*, *t*, *r*) with confidence intervals, effect sizes, degrees of freedom and *P* value noted<br>*Give P values as exact values whenever suitable.*
☒	☐	For Bayesian analysis, information on the choice of priors and Markov chain Monte Carlo settings
☒	☐	For hierarchical and complex designs, identification of the appropriate level for tests and full reporting of outcomes
☐	☒	Estimates of effect sizes (e.g. Cohen's *d*, Pearson's *r*), indicating how they were calculated

Our web collection on statistics for biologists contains articles on many of the points above.

Software and code

Policy information about availability of computer code

Data collection	All non-standard code can be found on figshare under the following link: https://figshare.com/s/0a88bd5d86238bd3e1b3
Data analysis	All statistical analyses were performed using GraphPad Prism (v6) or R Studio (v4.0.5).

For manuscripts utilizing custom algorithms or software that are central to the research but not yet described in published literature, software must be made available to editors and reviewers. We strongly encourage code deposition in a community repository (e.g. GitHub). See the Nature Portfolio guidelines for submitting code & software for further information.

Data

Policy information about availability of data

All manuscripts must include a data availability statement. This statement should provide the following information, where applicable:

- Accession codes, unique identifiers, or web links for publicly available datasets
- A description of any restrictions on data availability
- For clinical datasets or third party data, please ensure that the statement adheres to our policy

All raw sequencing data, as well as processed data files, including count matrices, and annotation sheets were uploaded to the gene expression omnibus and can be accessed with following accession code: GSE221202.
The methyl-array data was deposited on ArrayExpress and can be accessed under the accession number E-MTAB-13432.
Annotation sheets for Methylome analysis were downloaded from Ensembl (release 105).

GSEA was performed using genesets of MSigDB.
Sequencing reads were mapped against mouse transcriptome/genome (as specified in the methods) downloaded from Ensembl.
The TC-EC interactome mapping was performed using annotation sheets from CellPhoneDB.
Human CTC data was retrieved from publicly available datasets of References 34 and 35.

Human research participants

Policy information about studies involving human research participants and Sex and Gender in Research.

Reporting on sex and gender	not applicable
Population characteristics	not applicable
Recruitment	not applicable
Ethics oversight	not applicable

Note that full information on the approval of the study protocol must also be provided in the manuscript.

Field-specific reporting

Please select the one below that is the best fit for your research. If you are not sure, read the appropriate sections before making your selection.

☒ Life sciences ☐ Behavioural & social sciences ☐ Ecological, evolutionary & environmental sciences

For a reference copy of the document with all sections, see nature.com/documents/nr-reporting-summary-flat.pdf

Life sciences study design

All studies must disclose on these points even when the disclosure is negative.

Sample size	Sample size was determined based on prior experiments performed in the laboratory. For spontaneous metastasis models and experimental metastasis model using iECKO mice sample sizes were pre-determined using power analysis, assuming a 75% proportional standard deviation and a 60% difference in metastatic burden.
Data exclusions	For in vivo staining experiments with H-2Kd antibody, samples were excluded for which cells were not sufficently stained.<br>Mice were excluded that reached termination criteria prior to the experimetnal endpoint.
Replication	Biological replicates are clearly indicated for each figure panel and in the respective figure legends.
Randomization	Mice were randomly assigned to either control or treatment group.
Blinding	Data analysis after euthanasia was performed in a blinded fashion. Investigators were blinded to the group allocation of animals, except for experiments that involved the treatment of mice with LGK974 or LPS and experiments that required the continuous recording of animal body weight and tumor size (spontaneous metastasis experiment, long term experimental metastasis experiments). Here, blinding was not possible.

Reporting for specific materials, systems and methods

We require information from authors about some types of materials, experimental systems and methods used in many studies. Here, indicate whether each material, system or method listed is relevant to your study. If you are not sure if a list item applies to your research, read the appropriate section before selecting a response.

Materials & experimental systems

n/a	Involved in the study	
☐	☒	Antibodies
☐	☒	Eukaryotic cell lines
☒	☐	Palaeontology and archaeology
☐	☒	Animals and other organisms
☒	☐	Clinical data
☒	☐	Dual use research of concern

Methods

n/a	Involved in the study	
☒	☐	ChIP-seq
☐	☒	Flow cytometry
☒	☐	MRI-based neuroimaging

Antibodies

Antibodies used	Following antibodies were used:<br>CD31 APC (MEC13.3) 1:200 (0.2 mg/ml) BD Biosciences Cat# 551262, RRID:AB_398497;<br>CD31 PE (MEC13.3) 1:200 (0.2 mg/ml) BD Biosciences Cat# 553373, RRID:AB_394819;<br>CD31 BV786 (MEC13.3) 1:200 (0.2 mg/ml) BD Biosciences Cat# 740870, RRID:AB_2740522;<br>CD31 BV711 (MEC13.3) 1:200 (0.2 mg/ml) BD Biosciences Cat# 740680, RRID:AB_2740367;<br>CD45 APC (30-F11) 1:400 (0.2 mg/ml) BD Biosciences Cat# 559864, RRID:AB_398672;<br>CD45 PE (30-F11) 1:400 (0.2 mg/ml) BD Biosciences Cat# 553081, RRID:AB_394611;<br>CD45 BV786 (30-F11) 1:400 (0.2 mg/ml) BD Biosciences Cat# 564225, RRID:AB_2716861;<br>TER-119 APC (TER-119) 1:200 (0.2 mg/ml) BD Biosciences Cat# 557909, RRID:AB_398635;<br>TER-119 PE (TER-119) 1:200 (0.2 mg/ml) BD Biosciences Cat# 553673, RRID:AB_394986;<br>TER-119 BV785 (TER-119) 1:200 (0.2 mg/ml) BioLegend Cat# 116245, RRID:AB_2650921;<br>LYVE1 eFluor660 (ALY7) 1:200 (0.2 mg/ml) Thermo Fisher Scientific Cat# 50-0443-82, RRID:AB_10597449;<br>LYVE1 eFluor615 (ALY7) 1:200 (0.2 mg/ml) Thermo Fisher Scientific Cat# 42-0443-82, RRID:AB_10804146;<br>PDPN PE (eBio8.1.1) 1:200 (0.2 mg/ml) Thermo Fisher Scientific Cat# 12-5381-82, RRID:AB_1907439;<br>PDPN eFluor660 (eBio8.1.1) 1:200 (0.2 mg/ml) Thermo Fisher Scientific Cat# 50-5381-82, RRID:AB_11151516;<br>CD324 PE (DECMA-1) 1:200 (0.2 mg/ml) BioLegend Cat# 324106, RRID:AB_756068;<br>CD324 AF647 (DECMA-1) 1:200 (0.2 mg/ml) BioLegend Cat# 147308, RRID:AB_2563955;<br>H-2Kd APC (SF1-1.1) 5µg BioLegend Cat# 116620, RRID:AB_10645328;<br>H-2Kb/H-2Db AF647 (SF1-1.1) 5µg BioLegend Cat# 114612, RRID:AB_492931;<br>H-2Kd PE (SF1-1.1) 5µg BioLegend Cat# 116608, RRID:AB_313743;<br>mouse IgG2α, κ isotype APC (MOPC-173) 5µg BioLegend Cat# 400220, RRID:AB_326468;<br>mouse IgG2α, κ isotype PE (MOPC-173) 5µg BioLegend Cat# 400213, RRID:AB_2800438;<br>mouse IgG2α, κ isotype AF647 (MOPC-173) 5µg BioLegend Cat# 400234, RRID:AB_2864287;<br>CD31 (MEC13.3) 1:100 (15.625 µg/ml) BD Biosciences Cat# 550274, RRID:AB_393571;<br>Desmin (polyclonal) 1:100 (0.41 - 0.62 mg/ml) Abcam Cat# ab15200, RRID:AB_301744;<br>CD31 (polyclonal) 1:100 (0.2 mg/ml) R&D Cat# AF3628, RRID:AB_2161028
Validation	All antibodies used in this study were validated by the manufacturers for antigen specificity and species reactivity. The data can be found in the technical datasheets and attached references for each antibody. Antibodies used in this study showed specific staining consistent with published literature.

Eukaryotic cell lines

Policy information about cell lines and Sex and Gender in Research

Cell line source(s)	4T1, 4T1-GFP, D2.0R, D2A1-tom and E0771-GFP cells were gifts from the laboratories of Dr. Robert Weinberg (Whitehead Institute, Cambridge, MA), Dr. Jonathan Sleeman (Heidelberg University, Mannheim, Germany) and Dr. Kairbaan Hodivala-Dilke (Barts Cancer Institute, London, England), respectively. B16F10 and MDA-MB-231 cells were purchased from ATCC.
Authentication	Cell lines used were authenticated by morphology.
Mycoplasma contamination	Cell lines were routinely checked for mycoplasma contamination. For mycoplasma detection, 1 ml cell culture medium was centrifuged to remove cell debris and 2 µl of supernatant was directly used for detection PCR. PCRs for genotyping and mycoplasma detection were performed using REDTaq ReadyMix (Thermo Fisher Scientific) according to the manufacturer's instructions. PCR reactions were analyzed with the automated QIAxcel Advanced system according to the manufacturer's protocol (genotyping).
Commonly misidentified lines<br>(See ICLAC register)	No commonly misidentified lines were used in this study.

Animals and other research organisms

Policy information about studies involving animals; ARRIVE guidelines recommended for reporting animal research, and Sex and Gender in Research

Laboratory animals	Female NOD-SCID, BALB/c, and C57BL/6N mice were acquired from Janvier Labs. B6 Pdgfb-iCreERT2-IRES-EGFP x Wls floxed mice were bred in barrier animal facilities of the German Cancer Research Centre. Mice were housed in sterile cages, maintained in a temperature-controlled room and fed autoclaved water and food ad libitum. All animals were monitored daily for signs of disease and ear punches were used for genotyping the mice. Imported mice were allowed to acclimatise for a minimum of seven days before each experiment. For all experiments, 8-12 weeks old mice were used.
Wild animals	No wild animals were used in this study
Reporting on sex	For all experiments in this study female mice were used, due to the focus on breast cancer. To adhere to institutional 3R principles, male and female mice were used for genetic experiments using the experimental metastasis model.
Field-collected samples	No field-collected samples were used in this study.

Ethics oversight	All animal work was performed in accordance with German national guidelines on animal welfare and the regulations of the regional council Karlsruhe under permit numbers G-164/16, G-107/18, G-251/20, DKFZ305 and DKFZ370.

Note that full information on the approval of the study protocol must also be provided in the manuscript.

Flow Cytometry

Plots

Confirm that:

☒ The axis labels state the marker and fluorochrome used (e.g. CD4-FITC).

☒ The axis scales are clearly visible. Include numbers along axes only for bottom left plot of group (a 'group' is an analysis of identical markers).

☒ All plots are contour plots with outliers or pseudocolor plots.

☒ A numerical value for number of cells or percentage (with statistics) is provided.

Methodology

Sample preparation	Isolated lungs were minced on ice using curved serrated scissors. The minced tissue was resuspended in DMEM supplemented with Liberase Thermolysin Medium enzyme mix (0.2 mg/ml, Roche) and DNAse I (0.2mg/ml, Sigma Aldrich) and incubated at 37°C first for 15 min and then again for 12 minutes. After each incubation, minced tissues were passed through 18G cannula syringes 30 times. After the second incubation, digested tissues were passed through 100 µm cell strainer to remove tissue debris and cell clumps. The following steps were performed on ice. The digestion reaction was quenched by adding FCS and samples were centrifuged at 4°C and 400 g for 4 min. Erythrocytes were lysed by resuspending the cell pellet in pre-chilled 1x ammonium chloride potassium (ACK) buffer. The reaction was quenched by adding ice-cold PBS, followed by centrifugation. Whole lung single cell suspensions were passed through a 40 µm cell strainer and preincubated with anti-mouse CD16/CD32 Fc block (1:100, Thermo Fisher Scientific) for 15 min in flow buffer (PBS supplemented with 5% (vol/vol) FCS) and, subsequently, with the appropriate antibody-mix (see Suppl. Materials Table for detailed list of antibodies) for 20 min on ice.
Instrument	Cells were sorted using a BD bioscience Aria cell sorting platform (BD Biosciences) with 100 µm nozzle.<br>Samples were analysed using BD LSR Fortessa or BD FACSCanto II cell analyser, respectively.
Software	FACS Diva Software<br>FlowJo
Cell population abundance	Purity of bulk sorted fractions was estimated from singe cell experiments, which used the same gating strategy.<br>Lung EC fractions and TC showed purity of >99%, with macrophages and vascular mural cells being the main contaminants.
Gating strategy	All samples were gated on viable cells followed by exclusion of cell doublets and CD45+, LYVE1+, PDPN+ and TER119+.<br>EC were gated based on CD31 expression, TC based on reporter gene expression.<br>A detailed gating strategy is provided in Extended Data Fig. 1a, b and Extended Data Fig. 5 a-c.

☒ Tick this box to confirm that a figure exemplifying the gating strategy is provided in the Supplementary Information.
